# TraB family proteins are components of ER-mitochondrial contact sites and regulate ER-mitochondrial interactions and mitophagy

**DOI:** 10.1038/s41467-022-33402-w

**Published:** 2022-09-26

**Authors:** Chengyang Li, Patrick Duckney, Tong Zhang, Yanshu Fu, Xin Li, Johan Kroon, Geert De Jaeger, Yunjiang Cheng, Patrick J. Hussey, Pengwei Wang

**Affiliations:** 1 Key Laboratory of Horticultural Plant Biology (MOE), Huazhong Agricultural University, Wuhan, 430070 China; 2 Hubei Hongshan Laboratory, Wuhan, 430070 China; 3 Department of Biosciences, Durham University, South Road, Durham, DH1 3LE UK; 4 Department of Plant Biotechnology and Bioinformatics, Ghent University, Ghent, Belgium; 5 VIB Center for Plant Systems Biology, Ghent, Belgium

**Keywords:** Plant cell biology, Protein trafficking in plants, Mitophagy

## Abstract

ER-mitochondrial contact sites (EMCSs) are important for mitochondrial function. Here, we have identified a EMCS complex, comprising a family of uncharacterised mitochondrial outer membrane proteins, TRB1, TRB2, and the ER protein, VAP27-1. In Arabidopsis, there are three TraB family isoforms and the *trb1/trb2* double mutant exhibits abnormal mitochondrial morphology, strong starch accumulation, and impaired energy metabolism, indicating that these proteins are essential for normal mitochondrial function. Moreover, TRB1 and TRB2 proteins also interact with ATG8 in order to regulate mitochondrial degradation (mitophagy). The turnover of depolarised mitochondria is significantly reduced in both *trb1/trb2* and VAP27 mutants (*vap27-1,3,4,6)* under mitochondrial stress conditions, with an increased population of dysfunctional mitochondria present in the cytoplasm. Consequently, plant recovery after stress is significantly perturbed, suggesting that TRB1-regulated mitophagy and ER-mitochondrial interaction are two closely related processes. Taken together, we ascribe a dual role to TraB family proteins which are component of the EMCS complex in eukaryotes, regulating both interaction of the mitochondria to the ER and mitophagy.

## Abstract (web summary)

Contact sites permit the exchange of signals and materials between organelles. Here the authors characterize a protein complex associated with an ER-mitochondrial contact site in Arabidopsis that is required for mitochondrial function and mitochondrial turnover

## Introduction

In plant cells, organelles interact with each other to regulate multiple physiological activities^1–5^. Such intimate contact between the different organelles generates an interactive network that facilitates rapid material and signal exchange. The endoplasmic reticulum (ER) is physically connected to other membrane structures through membrane contact sites^6^ (MCS), including the ER-PM contact sites^7,8^ (EPCSs) and ER-mitochondrial contact sites^4,9^ (EMCSs). The EMCS interactions, in particular, are essential in multiple physiological activities, for example, Ca^2+^ transfer, lipid metabolism, autophagy, mitochondrial dynamics, and morphogenesis^3–5,9–11^. However, the protein components and biological relevance of EMCSs are poorly understood in plants compared to their counterparts in animals and fungi.

In order to maintain cellular homeostasis, impaired mitochondria have to be removed effectively through a process called mitophagy, which is a highly selective autophagic process. The successful removal of damaged mitochondria is able to reduce the production of excessive ROS, maintaining the oxidation-reduction environment and the stability of mitochondrial membrane potential^12–15^. Interestingly, the formation of autophagosomes (mitophagosomes) can also occur at EMCSs and it has been shown that altering the structure of EMCSs can reduce the number of autophagosomes produced^3,13,16^. Thus, it is likely that the establishment of ER-mitochondrial connections and the initiation of mitophagy are two inter-related processes.

Previous studies have shown that VAP27-1 localize to the ER-PM contact sites (EPCS) and the entire ER membrane^17–22^. VAP27-1 interacts with proteins involved in cytoskeleton interaction (e.g., NET3C), membrane trafficking (e.g., AtEH1/Pan, Clathrin), lipid transport (e.g., ORP3a), and lipid-droplet biogenesis (e.g., SEIPIN), regulating multiple subcellular activities. Here, we identify a mitochondrial membrane protein TRB1, which also interacts with VAP27-1 to regulate ER-mitochondrial association. In parallel, TRB1 also interacts with ATG8 to regulate mitochondrial degradation functioning as putative mitophagy receptors. From a combination of experimental approaches, our data indicate that TRB1-mediated EMCS participates in mitophagy, which in turn maintains healthy mitochondrial function, and energy homeostasis. These findings not only advance our knowledge of organelle interactions and mitophagy in general, but also bridge the gap in our understanding of how EMCSs and mitophagy are regulated *in planta*.

## Results

### TRB1 interacts with VAP27 at ER-Mitochondrial contact sites

In eukaryotic cells, the endoplasmic reticulum (ER) contacts with most membranes^2,7,20,23–27^. Similarly, the ER is also connected to the mitochondria through ER-Mitochondrial Contact Sites (EMCSs), which are essential in lipid metabolism, autophagy, mitochondrial function, and morphogenesis^3–5,11,28^. Across eukaryotes, the ER-integral membrane protein VAP27 (also known as VAP/Scs2) serves as a tethering factor between the ER and various organelles^17,18,29,30^. We, therefore, investigated whether VAP27 may mediate contact sites between the ER and mitochondria in plants. In *N. benthamiana* leaf epidermal cells co-expressing VAP27-1-GFP and the mitochondrial matrix marker, Mito-mCherry, we observed that some mitochondria partially co-localise with VAP27-1 on the ER surface (Supplementary Fig. 1a, arrow); such a phenomenon was not observed in control cells expressing the ER lumen marker, GFP-HDEL (Supplementary Fig. 1b). The ratio of overlapping mitochondria and ER was much higher in VAP27-1-expressing cells than the control cells (Supplementary Fig. 1c), suggesting VAP27-1 may play an important role in mediating ER-mitochondrial association in plants.

**Fig. 1:**
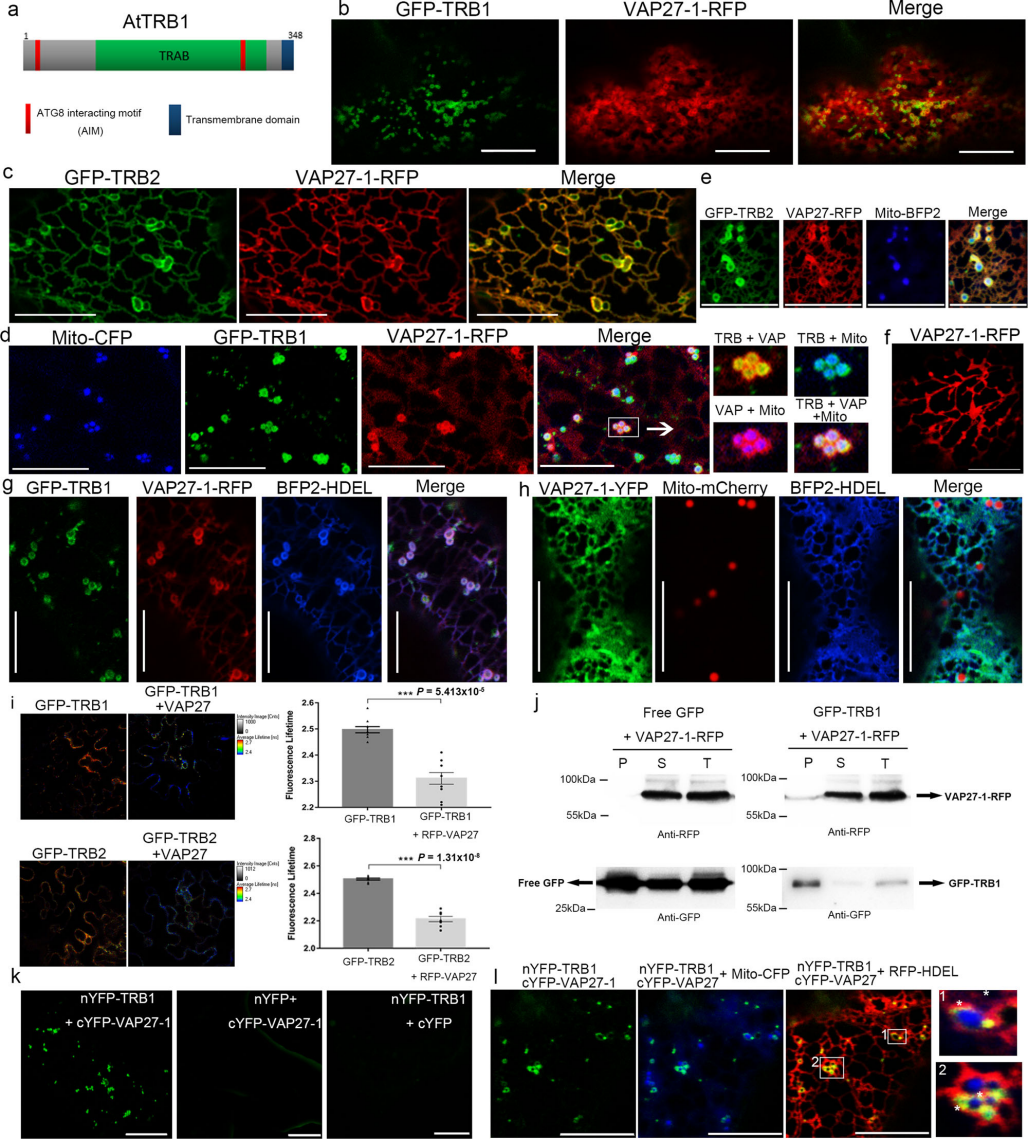
TRB1 and TRB2 proteins interact with VAP27 at ER-mitochondrial interface. **a** Schematic illustration of TRB1 protein in Arabidopsis; it contains two ATG8 interacting motifs (AIM), one TRAB homology domain and a C-terminal transmembrane domain. **b** GFP-TRB1 and VAP27-1-RFP co-localize at ER-derived donut-shaped membrane structures in *N. benthamiana* leaf epidermal cells. **c** GFP-TRB2 co-localizes with VAP27-1-RFP at the ER network and ring-shaped membrane structures. **d** GFP-TRB1 (green), VAP27-1-YFP (Red), and Mito-CFP (blue) co-expressed in *N. benthamiana* leaf epidermal cells, demonstrating that the ER derived donut-shaped structures superimpose with the mitochondrial outer membrane. **e** GFP-TRB2 (green), VAP27-1-YFP (Red), and Mito-mTAGBFP2 (blue) co-expressed in *N. benthamiana* leaf epidermal cells, the ER derived ring-shaped structures superimpose with the mitochondrial outer membrane. **f** Control cells expressing VAP27-1-RFP alone. **g**, **h** Triple expression of GFP-TRB1, VAP27-1-RFP, and mTAGBFP-HDEL in *N. benthamiana*. Recruitment of mTAGBFP2-HDEL-labelled ER to TRB1-VAP27-labelled hybrid structures was identified (**g**). However, this phenotype was not found in the absence of TRB1 (**h**), supporting the idea that TRB1-VAP27 interaction promotes ER-mitochondrial association. **i** FRET-FLIM further proved the interactions between TRB1/VAP27-1 and TRB2/VAP27-1. The fluorescence life-time of GFP-TRB1 (control) and GFP-TRB2 changes significantly (life-time reduces from 2.50 ± 0.01 to 2.31 ± 0.02 ns; and from 2.50 ± 0.01 to 2.21 ± 0.02 ns, respectively) in the presence of RFP-VAP27-1. **j** The interaction between VAP27-1 and TRB1 is confirmed by a GFP-Trap assay. VAP27-1-RFP was only pulled-down in the presence of GFP-TRB1 (right) but not with free GFP (left). P, pellet; S, supernatant; T, total. **k** Split-YFP based BiFC study showed cYFP-VAP27-1 and nYFP-TRB1 producing signals when co-expressed in *N. benthamiana*. **l** The YFP signal generated from VAP27-TRB1 BiFC colocalised with Mito-CFP and RFP-HDEL, demonstrating the interaction to be ER and mitochondrial-localised. Microscopy studies were repeated at least 3 times for every experiment. *n* = 10 independent biological samples for every FRET-FLIM analysis, error bars are SEM, *** *P* < 0.001 in two-tailed Student’s t tests (scale bar = 10 μm).

To screen for the tethering proteins through which VAP27 mediates EMCSs, TAP (Tandem affinity purification) tagging was performed using transgenic Arabidopsis cell cultures. After affinity purification followed by a MS proteomic screen, we identified TRB1 (Fig. 1a), a mitochondrial protein with unknown function, as an interacting protein of VAP27-1. There are two closely related TraB family proteins encoded by the Arabidopsis genome, TRB1 and TRB2, both of which co-localize with VAP27-1-RFP at numerous donut-shaped or ring-like structures when co-expressed (Fig. 1b, c). These structures are likely to be a hybrid membrane consisting of the mitochondrial membrane and the ER membrane because both the GFP-TRB1/TRB2 and VAP27-1-RFP signal co-localise at circular structures surrounding mitochondria (Fig. 1d, e). Next, we studied the cells expressing GFP-TRB1, VAP27-1-RFP, and mTAGBFP-HDEL, and found that the HDEL-labelled ER was also enriched at these donut-structures in the presence of TRB1 (Fig. 1g). This phenomenon is likely caused by a direct interaction between TRB1 and VAP27-1, as it is absent in cells only expressing VAP27-1 (Fig. 1f–h).

FRET-FLIM was used to further study these interactions. The fluorescence lifetimes of GFP-TRB1 and GFP-TRB2 are reduced significantly in the presence of RFP-VAP27-1, indicating physical interactions (Fig. 1i). Furthermore these in vivo interaction data were further confirmed using co-immunoprecipitation assays (Fig. 1j). In a BiFC assay, nYFP-TRB1 and cYFP-VAP27-1 generated YFP-fluorescent signals when co-expressed together (Fig. 1k), these signals were ER-associated and were found at the ER-mitochondrial interface (Fig. 1l). Taken together we conclude that TRB1 and VAP27-1 interact and that this interaction may serve to mediate the formation of EMCS.

### Phylogenetic analysis, expression, and localization of TraB family proteins

**Fig. 2:**
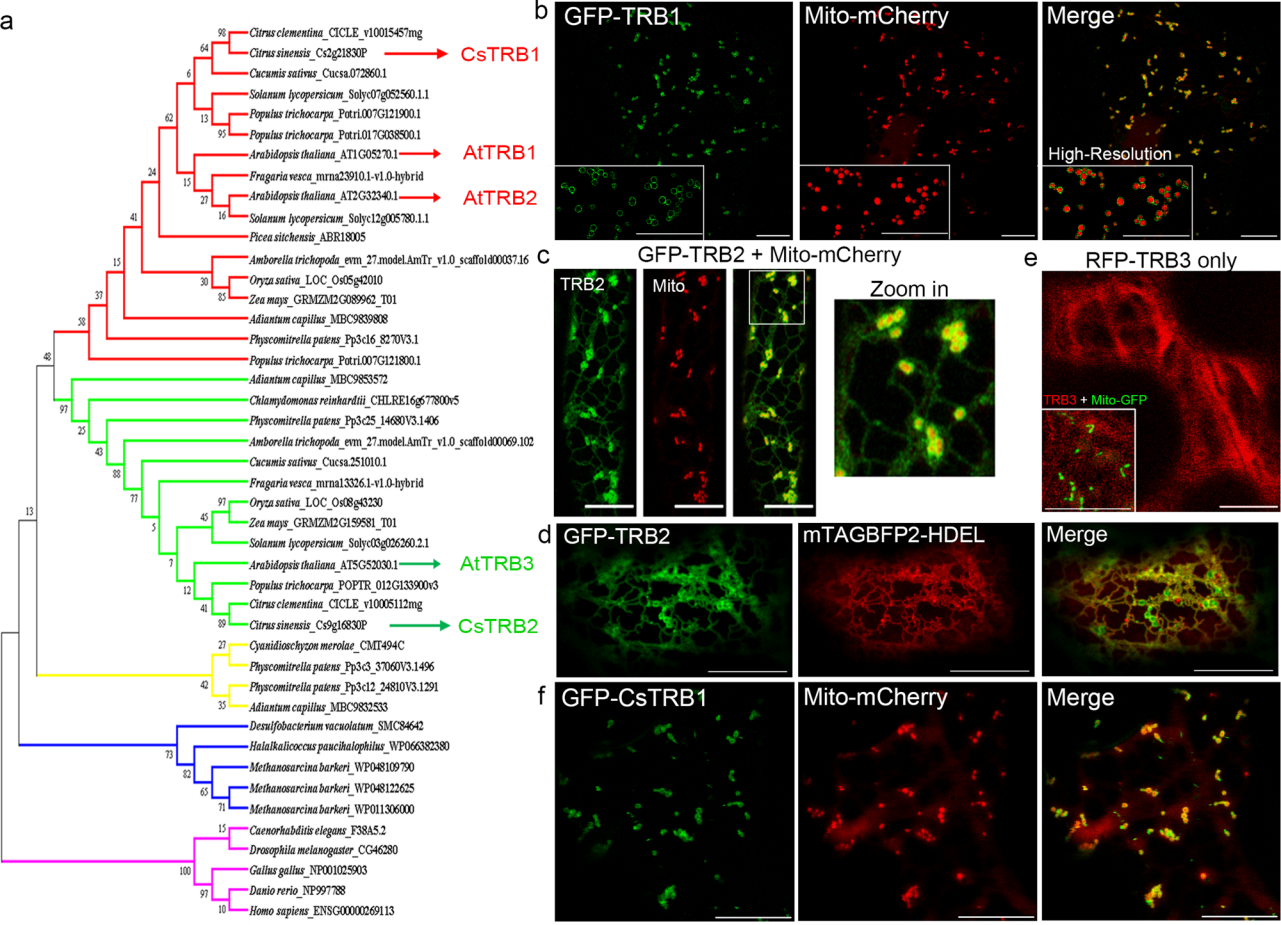
Phylogenetically conserved TraB family proteins localise to mitochondria and ER. **a** Phylogenetic tree of TraB family homologues showing that Arabidopsis TRB1 and TRB2 originate from the same clade, whereas TRB3 belongs to a separate clade that is more evolutionarily similar to their mammalian homologues. **b** In Arabidopsis, GFP-TRB1 localizes to the outer membrane of mitochondria that is labelled by the mitochondrial matrix marker, Mito-mCherry. The inset are additional images taken by high-resolution microscopy showing clear mitochondrial membrane localisation of TRB1. **c**, **d** GFP-TRB2 localizes to the outer mitochondrial membrane as well as the ER network (mTAGBFP2-HDEL labelled). **e** RFP-TRB3 mostly localizes to the cytoplasm, its signal is clearly distinct from the mitochondria maker. **f** In citrus, only one isoform of TraB family proteins containing TMD exists, which also localizes to the outer mitochondrial membrane in *N. benthamiana* leaf epidermal cells. Microscopy studies were repeated two times for **c** and at least three times for **b**, **d**–**f** (scale bar = 10 μm).

The TraB family proteins, TRB1 and TRB2 contain a C-terminal transmembrane domain, and a TraB-homology domain which currently has unknown function in eukaryotes (Fig. 1a). In Arabidopsis, TRB1 and TRB2 are highly conserved, whilst TRB3 is more divergent (Fig. 2a). GFP-TRB1 localises to ring-like structures that appear to be the outer membrane of mitochondria (OMM, Fig. 2b), while the subcellular localisation of TRB2, the closest homologue of TRB1 (sharing 73.4% peptide similarity), is found both at the ER network and the mitochondrial outer membranes (Fig. 2c, d). These results are consistent with previous observations from a proteomic analysis^31,32^, where it was found that endogenous TRB1 is enriched in mitochondria in both Arabidopsis, and citrus fruits. Plant TRB3 isoforms (which mainly localized to the cytoplasm, Fig. 2e) are more closely related to the mammalian and bacterial TraB family proteins, as none of them contain the predicted transmembrane domain that is found in TRB1, suggesting that this is likely to be an ancient form appearing earlier in evolution (Fig. 2a). The mitochondrial localisation of TRB1 is likely to be conserved in many higher plants, as the citrus homologue of TRB1, CsTRB1 also localised to the OMM (Fig. 2f).

TRB1 and TRB2 are expressed in most plant tissues with the strongest expression found in roots and flowers (Supplementary Fig. 2a, b). Under the control of their own promoters, expression of both GFP-TRB1 and GFP-TRB2 is further confirmed in vegetative tissues (Supplementary Fig. 2c–e). We found *pTRB1*:GFP-TRB1 localised to donut-shaped structures that are likely to be the outer-mitochondrial membrane, while *pTRB2*:GFP-TRB2 localised to donut and networked structures, reminiscent of the mitochondria and ER. As both exhibit similar expression profiles, sequence homology, and subcellular localisation, further studies were mainly focused on TRB1 to avoid repetition.

### TRB1 protein is important in mitochondrial movement, morphology, and energy metabolism

**Fig. 3:**
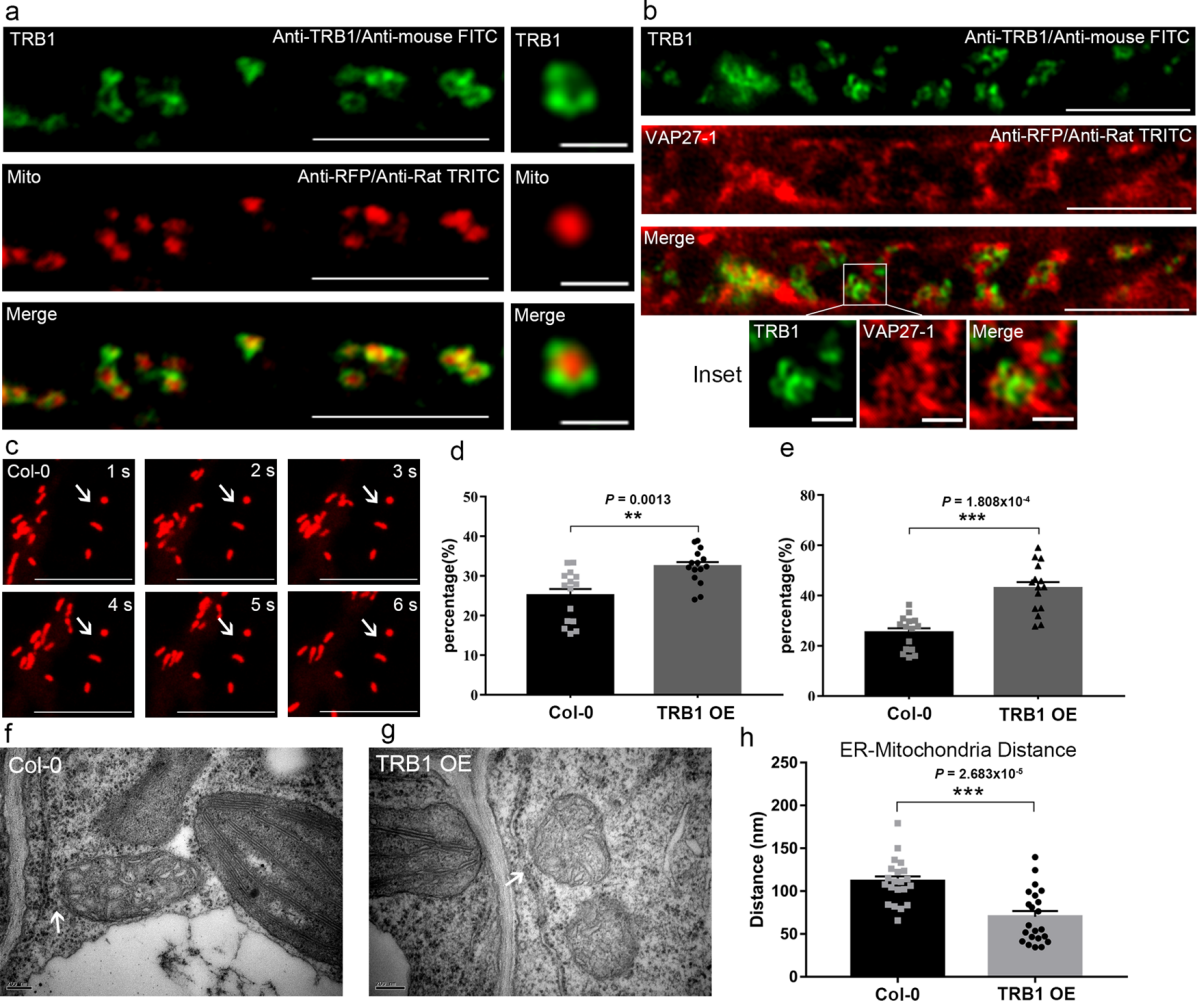
TRB1 protein is essential for mitochondrial movement and ER association. **a**, **b** Arabidopsis expressing Mito-mCherry and VAP27-RFP was immuno-labelled with a TRB1 antibody; the endogenous protein localized at the outer mitochondrial membrane, and in contact with VAP27-labelled ER membrane, respectively. **c** An example of stationary mitochondria (arrow) that stay immobile during the six second time course. **d**, **e** The percentage of stationary mitochondria (immobile for at least six seconds) increased significantly in the presence of GFP-TRB1 or VAP27-1. TEM study of wild type Arabidopsis (**f**) and stable Arabidopsis transformed with GFP-TRB1 (**g**), the distance between ER and mitochondria is significantly reduced compared to wild type plants (**h**). **a**, **b** Immunolabelling experiments were performed two times with similar results. **c**–**e** Microscopic imaging were repeated at least three times with similar results. For mitochondria movement analysis, *n* = 15 cells; For TEM analysis, at least 20 mitochondria from 15 cells in total examined over three biologically independent samples, error bars are SEM, ** 0.001 < *P* < 0.01, *** *P* < 0.001 in two-tailed Student’s t tests and with Welch’s correction in (**h**) (scale bar = 10 μm for light microscopy; scale bar = 500 nm for TEM).

Endogenous TRB1 is found localized to the outer mitochondria membrane and stays closely associated with VAP27 in root cells (Fig. 3a, b). This was demonstrated by immunofluorescence using an antibody raised against TRB1 (Supplementary Fig. 2f–g). These results confirm the live cell imaging data, suggesting that the endogenous TRB1 is also likely to facilitate the ER-mitochondrial association. In the presence of over-expressed GFP-TRB1 or VAP27-1-RFP, the percentage of stationary mitochondria (that stay immobile for more than 6 s, Fig. 3c) increased significantly, likely due to an increment of ER-mitochondria tethering (Fig. 3d, e). Enhanced ER-mitochondrial association at the ultrastructural level is observed in cells over-expressing GFP-TRB1 (Fig. 3f–h). Taken together, we have identified a EMCS complex comprising the ER-localised VAP27 and the OMM-localised TRB1.

When TRB1 is over-expressed in *N. benthamiana* leaf epidermal cells, the mitochondria became enlarged and swollen (Supplementary Fig. 3a), and the morphology of mitochondria changes from a tubular/oval shape to a spherical shape (Supplementary Fig. 3b). Such phenotypes are commonly observed when the functions of mitochondria are inhibited^33–35^. At the ultrastructural level, these mitochondria appear to be aggregated and more electron transparent, suggesting mitochondrial function is likely impaired (Supplementary Fig. 3c, d). TMRM stains the mitochondria according to their electron potential, which can be used as an indicator for mitochondrial activity. In cells treated with antimycin (a disruptor of ATP synthesis), a significant reduction of TMRM fluorescence is seen (Supplementary Fig. 3e, f). Interestingly, the TMRM signal is weaker in Arabidopsis stably over-expressing GFP-TRB1 (Supplementary Fig. 3g) in contrast to control plants expressing Mito-YFP (Supplementary Fig. 3h), indicating that the expression of GFP-TRB1 results in decreased mitochondrial membrane potential and activity (Supplementary Fig. 3i).

### The function of mitochondria is affected in the *trb1/trb2* mutant

**Fig. 4:**
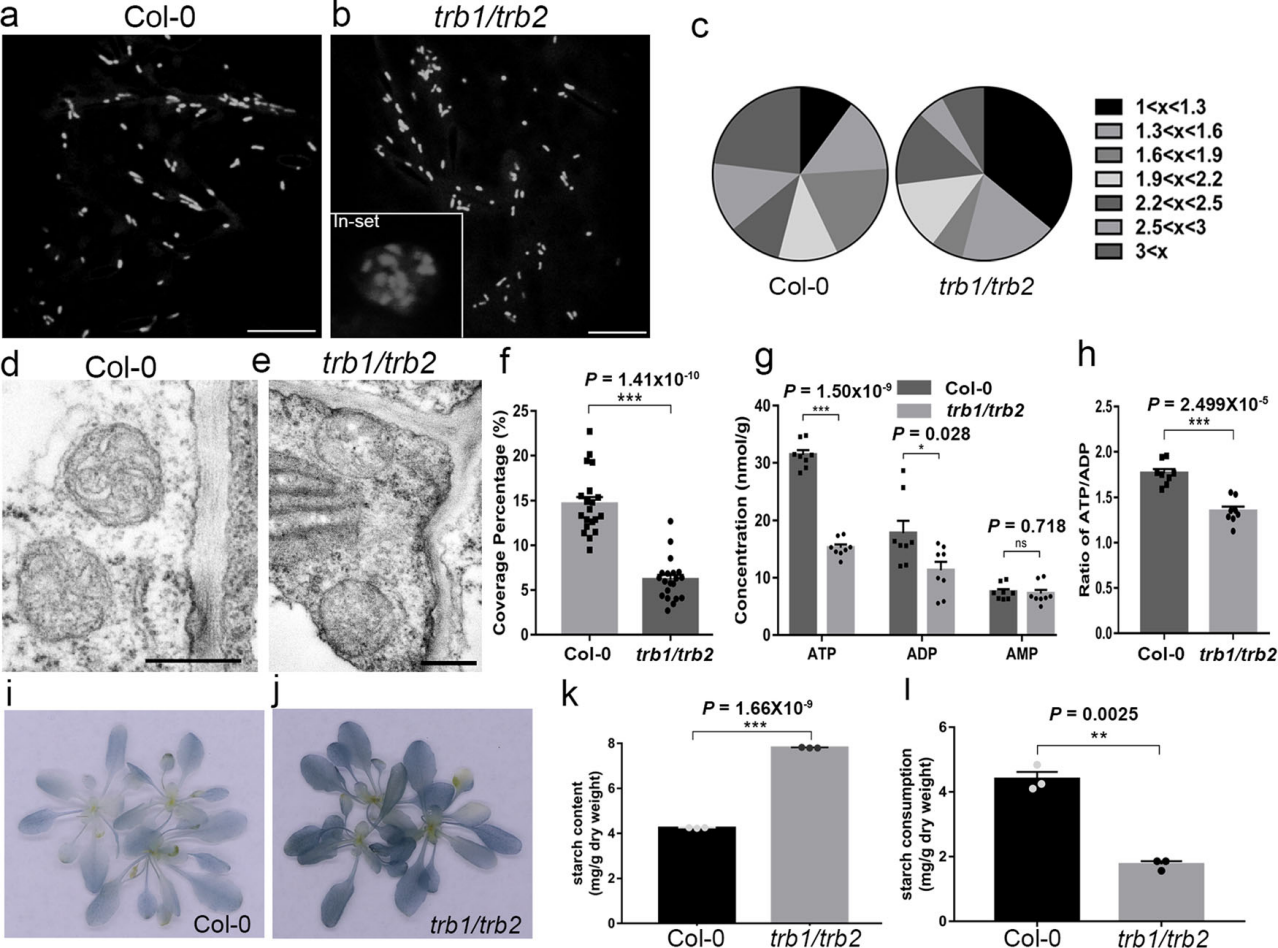
TraB family proteins are essential for normal mitochondrial structure and energy metabolism. **a**–**c** In the *trb1/trb2* T-DNA mutant Arabidopsis line stably expressing Mito-mCherry, the morphology of mitochondria is significantly altered, as indicated by the increased population of circular mitochondria (aspec ratio at 1-1.3, **c**). Mitochondrial aggregations (**b**, inset) were also frequently observed. In the *trb1/trb2* Arabidopsis, mitochondrial structures are also disrupted at the ultrastructural level (**e**); their internal membrane cisternae are more fragmented and the matrix becomes more electron-transparent compared with that in the wild type (**d**). **f** Quantification of the mitochondrial cristae coverage of *trb1/trb2* mutant and wild-type Arabidopsis. The levels of ATP, ADP and AMP are all significantly reduced in the *trb1/trb2* mutant (**g**), and the ATP/ADP ratio is also lower in the mutant (**h**), both results suggested a disruption in energy metabolism and mitochondrial function. **i**–**k** The starch levels in Col-0*, trb1/trb2* T-DNA plants (4–5 weeks old) were analysed by Lugol staining and the *trb1/trb2* loss-of-function mutant accumulated high levels of starch by the end of the light cycle. Such results were confirmed by spectrophotometry (**k**). **l** Under dark conditions, starch consumption is reduced in the *trb1/trb2* mutant, suggesting a reduction in mitochondrial respiration. *n* = 100 mitochondria from 10 cells for mitochondrial morphology analysis; *n* = 20 mitochondrial number over three biologically independent samples for TEM analysis; *n* = 9 biologically independent plants examined over three independent experiments for starch quantification; *n* = 8 biologically independent samples for ATP measurement, error bars are SEM, * 0.01 < *P* < 0.05, *** *P* < 0.001 in two-tailed unpaired t tests with Welch’s correction (scale bar = 10 μm for light microscopy; scale bar = 500 nm for TEM).

Considering the high possibility of functional redundancy, the T-DNA double mutant of *trb1/trb2* was generated to further study the function of TRB1 (Supplementary Fig. 4a, b). The structure and morphology of mitochondria were analysed using light and electron microscopy. In the *trb1/trb2* T-DNA mutants, a large proportion of the mitochondria show a spherical shape and become aggregated which is in contrast to that observed in the wild type (Fig. 4a–c). At the ultrastructural level, the structure of the mitochondria is also disrupted in the *trb1/trb2* mutants (Fig. 4d, e), with a significantly reduced coverage of the mitochondria inner cristae (Fig. 4f). Moreover, the total ATP level and the ATP/ADP ratio are reduced significantly in the double mutant (Fig. 4g, h), indicating an aberrant energy metabolism.

In plants, starch is an energy reserve and its level in plants is closely related to energy homeostasis^36,37^. In agreement with the energy metabolism defect, we found strong starch accumulation in the *trb1/trb2* mutant (Fig. 4i–k). In order to eliminate the influence from photosynthesis, and observe starch consumption directly related to mitochondrial activity, we further analysed the change in starch levels under dark conditions. We found a decrease in the reduction of the starch level in the *trb1/trb2* mutants compared to the wild type (Fig. 4l), suggesting starch consumption is inhibited. Meanwhile, another independent *trb1/trb2* double mutant was also created using CRISPR/Cas9 (Supplementary Fig. 5a, b) and a similar starch phenotype was observed (Supplementary Fig. 5c, d). In summary, these observations are consistent with the functions of TRB1 in regulating mitochondrial activity and ER-mitochondrial interaction, since mitochondrial function and integrity are regulated by EMCSs^38–41^.

### TRB1 interacts with ATG8 through the conserved AIM motif

**Fig. 5:**
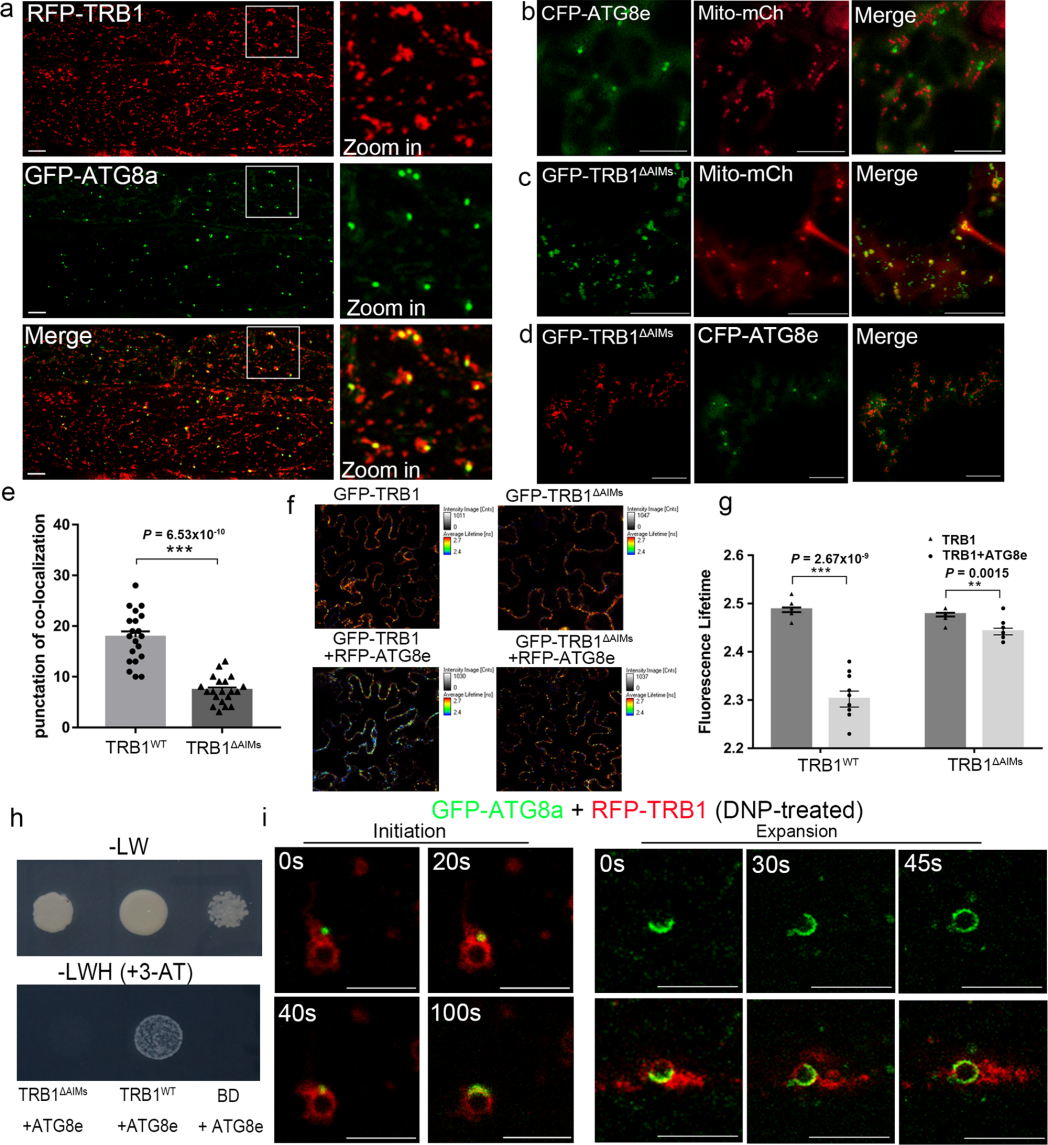
TRB1 interacts with ATG8 through the ATG8 interacting motif (AIM). **a** In Arabidopsis stably transformed with RFP-TRB1 and GFP-ATG8a under non-stressed conditions, GFP-ATG8a positive structures are recruited to the mitochondria that are labelled with RFP-TRB1 in hypocotyl epidermal cells. **b** Such ATG8e-mitochodrial co-localization is rarely seen in cells expressing a mitochondrial maker, Mito-mCherry. *N. benthamiana* leaf epidermal cells expressing GFP-TRB1^ΔAIMs^ (TRB1 without the ATG8 interacting motif) with a mitochondrial marker (**c**) and an autophagosome marker (**d**). GFP-TRB1^ΔAIMs^ still localizes to the mitochondrial outer membrane but did not co-localise with ATG8. **e** Quantifications of TRB1 and ATG8-labelled autophagosomes in *N. benthamiana* leaf epidermal cells; the numbers of autophagosomes that associate/co-localise with TRB1 per field (80 µm × 15 µm) was measured. **f**, **g** FRET-FLIM further proved that the interactions between TRB1 and ATG8 is reliant on the AIM motifs. The fluorescence life-time of GFP-TRB1 (control) changes significantly (life-time reduces from 2.49 ± 0.01 to 2.30 ± 0.02 ns) in the presence of RFP-ATG8e, while the fluorescence life-time of GFP-TRB1^ΔAIMs^ (control) only dropped by 0.04 ns in the presence of RFP-ATG8e; such a difference is too little to indicate an interaction. **h** Using a one-on-one yeast-two-hybrid assay, ATG8e was confirmed to interact with full length TRB1 but not with the TRB1^ΔAIMs^ mutant. **i** Following DNP-induced mitochondrial depolarization in Arabidopsis root cells expressing GFP-ATG8a and RFP-TRB1, the ATG8a labelled autophagosome membrane was recruited to a TRB1-labelled swollen mitochondrion. The two set of images are from independent studies showing different stages of mitophagy biogenesis. *n* = 10 biologically independent samples for every FRET-FLIM analysis, *n* = 20 cells examined over three independent experiments for ATG8 co-localisation analysis, error bars are SEM, ** 0.001 < *P* < 0.01, *** *P* < 0.001 in two-tailed Student’s t tests in (**e**) and Mann Whitney test in **g** (scale bar = 10 μm).

As the molecular functions of TRB1 are yet uncharacterised, we performed further sequence analysis and we found two ATG8-interacting motifs (AIM) in TRB1 protein (Supplementary Fig. 6a), suggesting it may interact with ATG8 and participate in the autophagy pathway^42,43^. Therefore, stable Arabidopsis lines expressing GFP-ATG8a and RFP-TRB1 were generated. We found that ATG8a signal is recruited to TRB1 labelled mitochondria under normal growth conditions, with strongest co-localisation found in hypocotyl epidermal cells (Fig. 5a); such co-localisation is rarely found in cells expressing CFP-ATG8e and Mito-mCherry (Fig. 5b).

Next, we mutated the putative AIM motifs of TRB1 (denoted as TRB1^ΔAIMs^, Supplementary Fig. 6a). In *N. benthamiana* leaf epidermal cells, GFP-TRB1^ΔAIMs^ localizes to the mitochondria like the full-length protein (Fig. 5c). However, its co-localization with ATG8 was found to be significantly reduced, in contrast to cells expressing wild-type TRB1 (Fig. 5d, e). FRET-FLIM was used to study the interaction of TRB1^ΔAIMs^ with ATG8 (Fig. 5f, g). We observed that the fluorescence lifetimes of GFP-TRB1 are reduced significantly in the presence of RFP-ATG8e, indicating physical interactions, whereas the GFP lifetimes exhibit little reduction when GFP-TRB1^ΔAIMs^ is expressed with RFP-ATG8e using the same conditions (Fig. 5g), indicating that GFP-TRB1^ΔAIMs^ does not interact with RFP-ATG8e. Similarly, co-localization and interaction between TRB2 and ATG8 was confirmed in vivo (Supplementary Fig. 6b, c). Using one-on-one yeast-two-hybrid assays, the yeast strains only grew on selective medium when co-transformed with non-mutated TRB1 and ATG8, but were not able to grow when co-transformed with TRB1^ΔAIMs^ and ATG8 (Fig. 5h). These data indicate that the AIM motif is essential for the interaction between TRB1 and ATG8e.

Furthermore, we demonstrated an interaction between TRB1 and ATG8 using the recently established Knocksideways in Plants (KSP) system^44^. This assay utilises the well-characterised, rapid rapamycin-dependent heteromerisation of FKBP (FKBP domain of HsFKBP12) and FRB (FKBP12 rapamycin-binding domain of mTOR) to induce rapid delocalisation of an FBKP-tagged bait construct and prey interactors to an FRB-tagged anchor. FKBP-mCherry-TRB1^WT ΔTMD^ was expressed alongside the prey construct, YFP-ATG8e, and the previously described microtubule-localised anchor construct, EB1a-mTAGBFP2-FRB^44^ in *N. benthamiana* leaf epidermal cells. Following rapamycin treatment, YFP-ATG8e was pulled from the cytosol to the microtubule network together with FKBP-mCherry-TRB1 indicating a physical interaction, consistent with previously observed interactions using KSP^44^ (Supplementary Fig. 7a, c). Conversely, no such interaction was observed when an FKBP-mCherry-TRB1^ΔAIMs ΔTMD^ bait construct with mutant AIM domains was used as prey, and YFP-ATG8e was not pulled to the microtubule cytoskeleton (Supplementary Fig. 7b, d). The data further demonstrates an interaction between TRB1 and ATG8, dependent on the AIM domains, and also indicates that TRB1 interacts with cytosolic ATG8, perhaps functioning to recruit ATG8 from the cytosol to the mitochondrial membrane. We therefore proceeded to investigate whether cytosolic ATG8 could be recruited to TRB1 at the mitochondrial membrane in vivo.

It is known that DNP promotes mitochondrial depolarisation and mitophagy^45–47^. After DNP treatment, time lapse confocal microscopy revealed the dynamic recruitment and expansion of ATG8 labelled autophagosome membranes to the swollen mitochondria in the presence of TRB1 (Fig. 5i). This is consistent with a role for TRB1 in its interaction with ATG8 and recruitment of ATG8 to the mitochondrial membrane during mitophagy.

### The turnover of TRB1 is dependent on the autophagy pathway

**Fig. 6:**
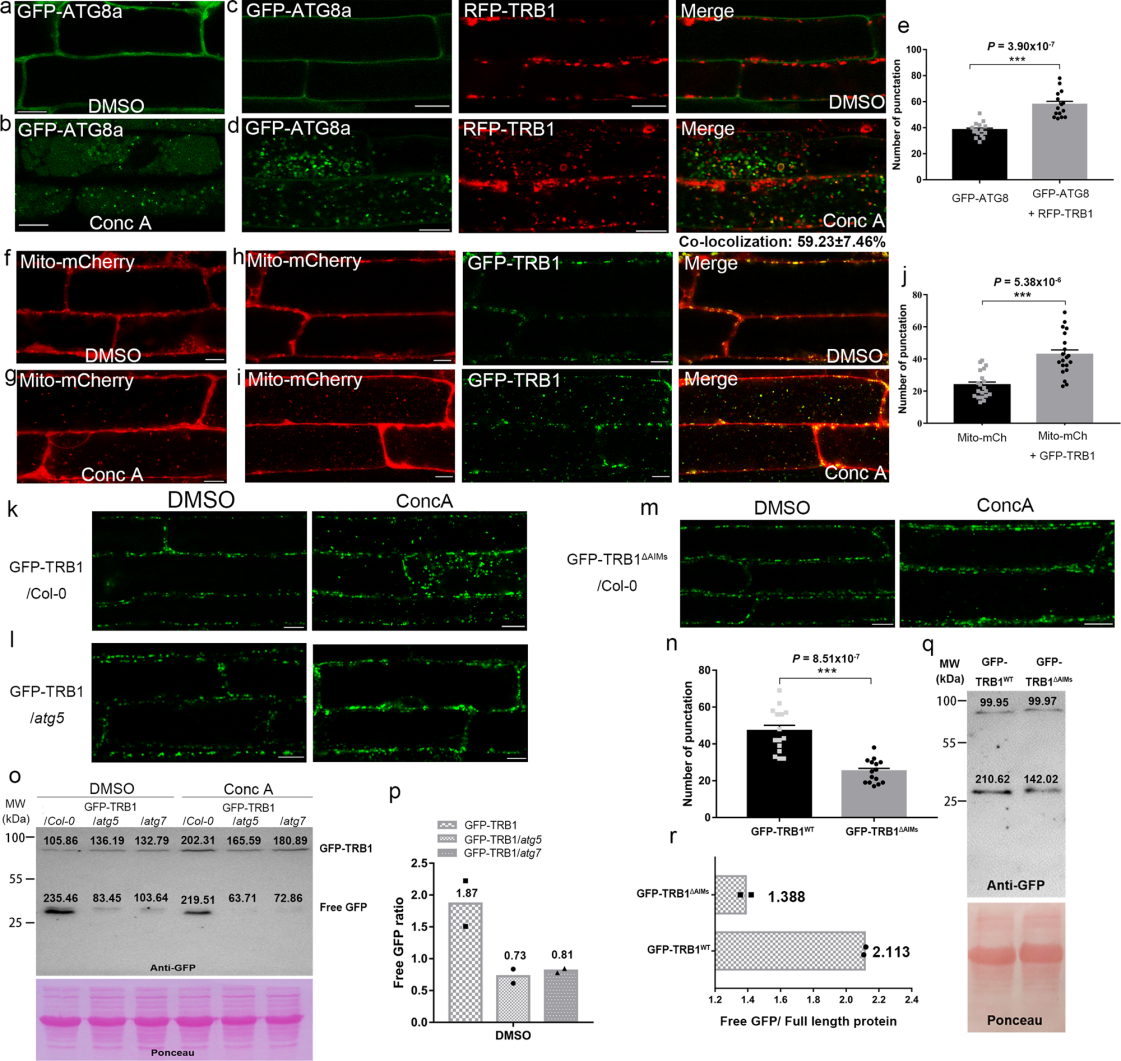
TRB1 over-expression promotes autophagy flux and the degradation of mitochondria through the autophagy machinery. **a**–**d** Arabidopsis plants expressing GFP-ATG8a or GFP-ATG8a + RFP-TRB1 grown in MS growth media without stress. After Conc A treatment, GFP-ATG8a labelled autophagosome structures were identified inside the vacuole (**b**); autophagosomes that contain mitochondrial signal (labelled by RFP-TRB1) are also abundant in cells expressing GFP-ATG8a and RFP-TRB1 (**d**). In contrast, little vacuole signal was seen in cells treated with DMSO as the control (**a**, **c**). **e** Quantifications of autophagosomes numbers in the vacuole per field (80 µm × 15 µm) after conc A. **f**–**i** Arabidopsis plants expressing Mito-mCherry or Mito-mCherry + GFP-TRB1 grown in MS growth media without stress. After Conc A treatment, numerous mitochondria (either labelled with Mito-mCherry or co-labelled with Mito-mCherry + GFP-TRB1) accumulated in the vacuole (**g**, **i**). In contrast, little vacuole signal was seen in cells treated with DMSO as the control (**f**, **h**). **j** The number of vacuole-accumulated mitochondria per field (80 µm × 15 µm) increased in cells over-expressing TRB1 after Conc A treatment. **k**, **l** GFP-TRB1 localization in wild type and an autophagy deficient mutant (*atg5*) under non-stressed conditions upon Conc A treatment. No punctate structures are found to accumulate inside the vacuole of cells in the *atg5* mutant, indicating that the transport of TRB1-labelled mitochondria into the vacuole relies on the core autophagy machinery. Arabidopsis expressing GFP-TRB1^ΔAIMs^ were treated with Conc A (**m**), the vacuole-accumulation of GFP positive signal is significantly reduced in comparison with Arabidopsis expressing non-mutated GFP-TRB1 (**n**). **o**, **p** Western blot analysis of GFP-TRB1 levels in Col-0, *atg5* and *atg7* Arabidopsis seedlings with and without Conc A treatment (**o**), the result shows a reduced level of degradation of GFP-TRB1 in both of the autophagy defective mutants (the band intensity is shown in grey scale), as indicated by the reduction in free GFP levels and ratio of free GFP/Full-length protein (**p**). **q**, **r** Western blot analysis of the protein levels of GFP-TRB1 and GFP-TRB1^ΔAIMs^ in Arabidopsis seedlings under non-stressed condition (**q**, the band intensity is shown in grey scale), the result shows that the degradation of GFP-TRB1^ΔAIMs^ is reduced, as indicated by the reduction in free GFP level and ratio of free GFP/Full-length protein (**r**). *n* = 15 (**e**), 20 (**j**) and 15 (**n**) cells examined over three independent experiments for image quantification respectively; *n* = 2 for every western blot analysis, error bars are SEM, *** *P* < 0.001 in two-tailed Student’s t tests and with Welch’s correction in **j** (scale bar = 10 μm).

Concanamycin A (ConcA) is a V-ATPase inhibitor that inhibits lytic vacuole activities and induces autophagosome accumulation (Fig. 6a, b)^48^ and we have used it to determine the autophagy flux of GFP-TRB1 under different conditions. In lines co-expressing RFP-TRB1 and GFP-ATG8a, large numbers of the TRB1 labelled puncta co-localize with ATG8 (59.23 ± 7.46%, Fig. 6c, d) after Conc A treatment, suggesting that TRB1 is targeted to the vacuole with the autophagosomes. The number of vacuole-accumulated autophagosomes increases significantly in the presence of RFP-TRB1 (Fig. 6e). Similarly, in cells expressing GFP-TRB1 and Mito-mCherry, both proteins co-localize in the vacuole after Conc A treatment (Fig. 6f–i), and the number of vacuole-accumulated mitochondria increased significantly (Fig. 6j) in contrast to the control cells only expressing Mito-mCherry (Fig. 6f, g). These data suggest a possible function for TRB1 in promoting autophagy/mitophagy. Furthermore, in transgenic Arabidopsis expressing both GFP-TRB2 and Mito-mCherry, strong co-localisation and vacuole accumulation of these proteins was found after Conc A treatment (Supplementary Fig. 6e, f).

The internalization of GFP-TRB1 to the vacuole is dependent on autophagy, as no vacuole accumulation of TRB1 is found after Conc A treatment of the same GFP-TRB1 transgenic line crossed into either the *atg5* and *atg7* autophagy deficient mutants (Fig. 6k, l). In parallel, we generated Arabidopsis transgenic lines expressing the ATG8 binding-defective GFP-TRB1^ΔAIMs^ mutant construct, in which less GFP-TRB1^ΔAIMs^ vacuole accumulation is observed following Conc A treatment (Fig. 6m, n). Therefore, these results suggest that the vacuole internalization of TRB1 is dependent on both the interaction with ATG8 and a functional autophagy pathway.

To further confirm the autophagic degradation of TRB1, protein levels of GFP-TRB1 in autophagy-deficient backgrounds was monitored using western blotting^48^. The levels of full-length protein and their degradation products (free GFP cleaved from the fusion protein) were analysed. As expected, the level of full-length GFP-TRB1 protein was found to be higher in *atg5, atg7* mutants under normal growth conditions, whilst the level of free GFP resulting from protein degradation was much lower compared to the Col-0, indicating impairment of TRB1 degradation in the autophagy mutants (Fig. 6o). When treated with Conc A, the protein levels of GFP-TRB1 accumulated to a higher level in the wild type (as shown by the reduced ratio of free GFP/full-length protein, Fig. 6p), indicating that blocking autophagic degradation prevents TRB1 degradation. Meanwhile, Conc A treatment of both *atg5* and *atg7* mutants did not result in a pronounced accumulation of GFP-TRB1 levels (Fig. 6o, p). Transgenic plants expressing GFP-TRB1^ΔAIMs^ were also analysed and we found that the intensity ratio of free GFP/full-length protein is reduced significantly in GFP-TRB1^ΔAIMs^ plants (Fig. 6q, r), indicating the impaired degradation of TRB1 when its interaction with ATG8 is inhibited.

Interestingly, the impairment of autophagic degradation of GFP-TRB1 also resulted in developmental defects. Arabidopsis plants expressing GFP-TRB1^ΔAIMs^ are also dwarfed (Supplementary Fig. 8), indicating that the autophagy-dependent protein turnover of TRB1 is essential for normal plant development, and over-accumulation may lead to mitochondrial dysfunction (Supplementary Fig. 3a–d). Taken together, in addition to the function as an ER-Mitochondrial-association factor by its interaction with VAP27-1, TRB1 and TRB2 proteins also interact with ATG8 and are degraded via the autophagy pathway. However, whether TraB family proteins are part of the machinery for mitophagy, or simply acts as a cargo for autophagic degradation is the next question to be determined.

### Stress-induced mitophagy is affected in the *trb1/trb2* mutant

**Fig. 7:**
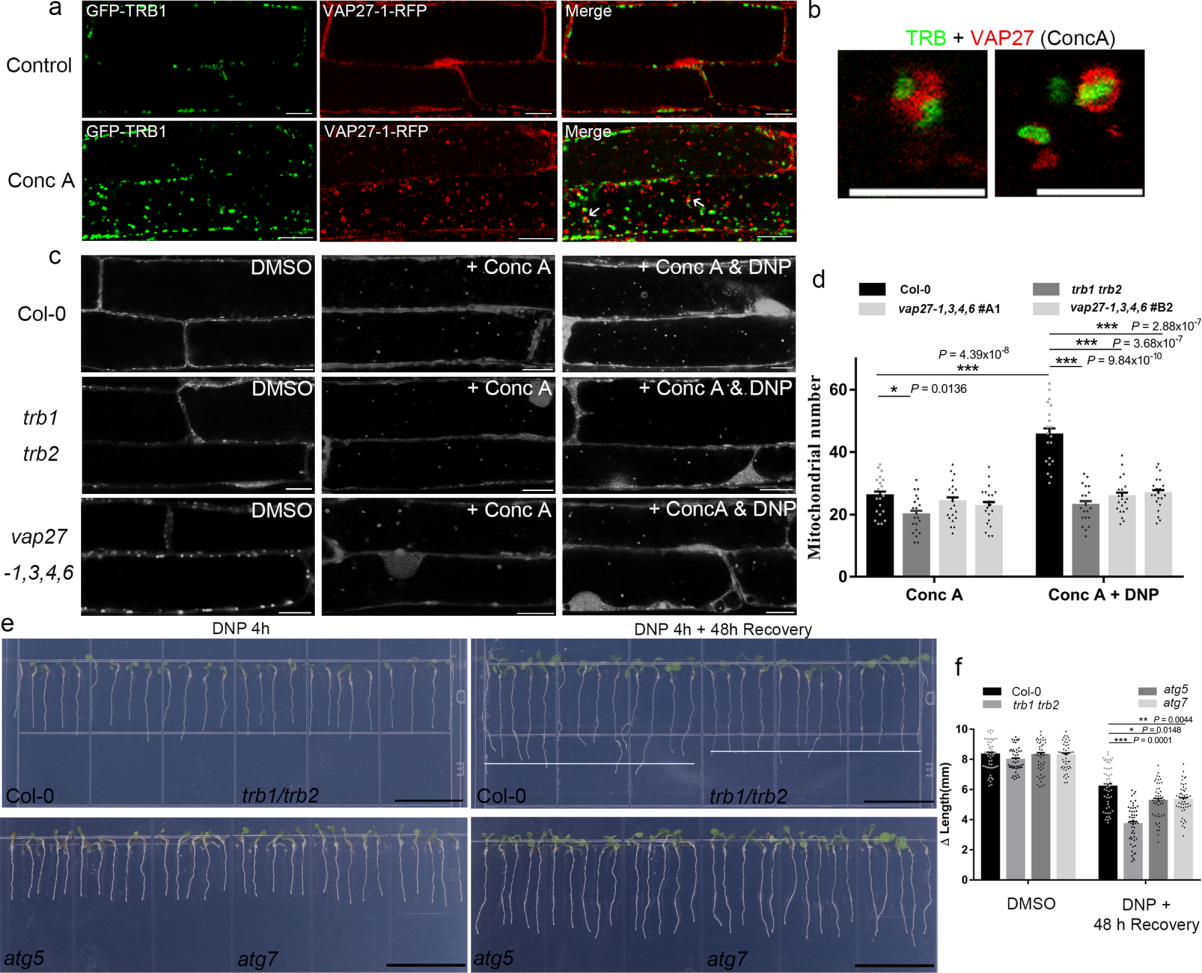
Mitochondrial stress-induced mitophagy is inhibited in the *trb1/trb2* mutants. **a** Transgenic Arabidopsis expressing GFP-TRB1 and VAP27-1-RFP were treated with Conc A. The majority of TRB1-labelled mitochondria are found in contact with VAP27-labelled ER membrane inside the vacuole, suggesting that these two structures are internalised together. **b** High magnification images of (**a**). **c**, **d** Arabidopsis plants (Col-0; *trb1/trb2*; *vap27-1,3,4,6*) stably expressing Mito-mCherry were treated with DNP and Conc A to study the activity of mitophagy. Under non-stressed conditions, the mitochondrial turnover rate is similar in all plants analyzed. However, the number of vacuole-accumulated mitochondria is significantly reduced in both the *trb1/trb2* and the *vap27-1,3,4,6* (two independent lines, A1 and B2) mutants treated with DNP, suggesting that an interruption of ER-Mitochondrial interaction affects mitochondrial turnover following mitochondrial stress/mitochondrial damage. **e**, **f** The recovery of Arabidopsis seedlings after 4 h of DNP treatment was studied. The root growth of the *trb1/trb2* mutant plants is significantly reduced compared to that in the wild type, suggesting that the DNP induced mitochondrial dysfunction has more impact on plant development when the function of TRB1 and TRB2 is impaired. The change of root length before and after recovery was measured, denoted as Δlength. *n* = 20 cells examined over three independent experiments for confocal quantification; *n* = 90 independent plants for root analysis; error bars are SEM, * 0.01 < *P* < 0.05, ** 0.001 < *P* < 0.01, *** *P* < 0.001 in two-way ANOVA, Sidak’s multiple comparisons test in **d** and Dunnett’s multiple comparisons test in **f** (scale bar = 10 μm for confocal; Scale bar = 200 nm for TEM; scale bar = 15 mm for root analysis).

It is known in yeast and mammalian systems that the ER-Mitochondrial interfaces are essential for autophagosome biogenesis and mitophagy^16,49,50^, so it is also reasonable to propose that TRB1-VAP27 mediated ER-Mitochondrial interaction and TRB1-ATG8 regulated mitophagy are two closely related processes. Transgenic Arabidopsis expressing GFP-TRB1 and VAP27-RFP were treated with Conc A. A large number of the TRB1 labelled mitochondria are found in close contact with VAP27 labelled ER membrane after internalization to the vacuole, suggesting that ER and Mitochondria likely stay in contact during mitophagy (Fig. 7a, b).

To investigate whether TRB1 and VAP27 are important for regulating mitophagy, we examined mitophagy activity in their mutant plants. Mito-mCherry was transformed into wild type Arabidopsis and the *trb1/trb2* double mutant, and the level of mitophagy flux (indicated by the number of internalized mitochondria) was measured after Conc A treatment. Under normal growth conditions, the number of vacuole-internalized mitochondria was found to be similar in both Col-0 and *trb1/trb2* but a significant reduction was observed in the *trb1/trb2* mutant when these plants were subjected to DNP-induced mitochondrial depolarization (Fig. 7c). This result indicates that TraB family proteins are needed for stress-induced mitophagy above the normal basal levels. In addition, two independent *vap27-1,3,4,6* quadruple mutants were generated (Supplementary Fig. 9a) using CRISPR/Cas9 with the intention of disrupting the VAP27-TRB1 interaction at EMCSs, as these ER localized VAP27s exhibit partial co-localization with TRB1 (Supplementary Fig. 9b–d). Both mutants exhibit defects in stress-induced mitophagy similar to the *trb1/trb2* mutant (Fig. 7c, d).

We hypothesise that heightened levels of impaired mitochondria (TMRM negative) resulting from DNP-induced depolarisation (Supplementary Fig. 10a) requires mitophagy to maintain mitochondrial homeostasis, in which TRB1-VAP27 plays an important role. Significant accumulation of depolarized mitochondria is found in the loss-of-function mutants of *trb1/trb2* and *vap27-1,3,4,6* (Supplementary Fig. 10b, c), as indicated by the proportion of cytoplasmic mitochondria with reduced TMRM signals (Supplementary Fig. 10c). In conclusion our data demonstrate that TRB1: 1. is OMM-localized; 2. interacts with ATG8; 3. is transported to the vacuole using the autophagy machinery; 4. over-expression enhances mitophagy and furthermore, 5. in *trb1/trb2* knock out mutants, DNP induced mitophagy is partially impaired. These data favour the hypothesis that TRB1 is a mitophagy receptor, as well as performing a function in ER-Mitochondrial interaction.

Mitochondrial damage releases reactive oxygen species and other toxic compounds^15,51^, which induces cell stress. Therefore, unsuccessful removal of damaged/depolarised mitochondria may have negative impacts on plant growth^52,53^. To test this hypothesis, the Arabidopsis *trb1/trb2* and *vap27-1,3,4,6* mutants were subjected to acute DNP treatments and their growth was analysed 2 days after recovery on DNP-free media. We observed that *trb1/trb2* and *vap27-1,3,4,6* mutant plants exhibit relatively weak recovery compared to Col-0 plants after 2 days (Fig. 7e, f and Supplementary Fig. 11), suggesting that both mutants are defective in the maintenance of mitochondrial homeostasis, which we attribute to defects in mitophagy. This observation is consistent with our previous observations that impairment of TRB1-promoted mitophagy (through overexpression of GFP-TRB1^ΔAIMs^) results in growth defects (Supplementary Fig. 8), and demonstrates the importance of endogenous TRB1 in plant mitophagy. As the positive control, the autophagy defective mutants (*atg5* and *atg7*) were treated with DNP and studied in the same way; reduced recovery was also prominent (Fig. 7e, f). However, the growth perturbation of the *trb1/trb2* mutant after DNP treatment is more severe than the same treatment of the known autophagy mutants, and this is likely because TRB1 may regulate multiple mitochondrial activities in addition to mitophagy.

## Discussion

It is known that ER-Mitochondrial interactions are essential for mitochondrial division and signal/substrate exchange between the two compartments. In animal cells, the protein composition of EMCSs has been extensively studied in the last decade, and proteins such as MFN, Miro, and dynamin-related proteins are known to have important functions in regulating mitochondrial function and morphogenesis^11,35,41,49,54,55^. In contrast, only a few plant proteins have been suggested to regulate ER-mitochondrial interaction, such as Miro2 and MELL1, which have been studied in tobacco and *Physcomitrella patens*, respectively^11,56^. In this study, we have identified the evolutionarily conserved TraB family proteins as regulators of two putative interrelated pathways. Firstly, they interact with ER-localized VAP27 to regulate ER-Mitochondrial interactions, and secondly, they interact with ATG8 to regulate mitochondrial degradation working as putative mitophagy receptors. Our data indicates that TRB1 has important role in mitochondrial homeostasis through the regulation of mitophagy, which is essential for normal plant growth under stress conditions.

**Fig. 8:**
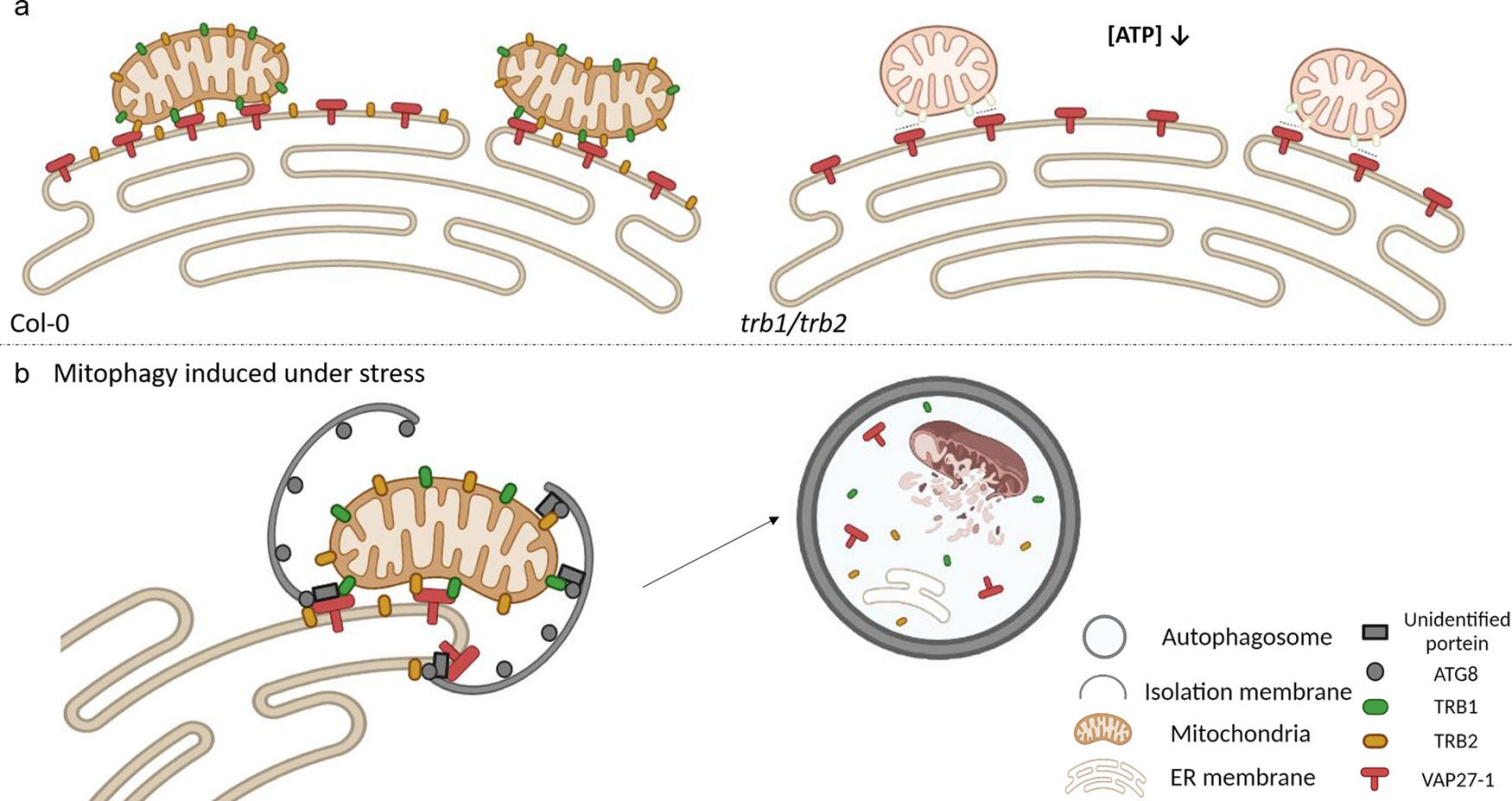
Proposed model for TRB1-VAP27 mediated Mitochondria-ER interaction and mitophagy. **a** Under normal conditions, mitochondria are tethered to the ER through the TRB1-VAP27 complex at EMCSs; this link is essential for mitochondrial function (e.g., movement, morphology, and energy metabolism). **b** TRB1 also interacts with ATG8, a function that is essential for the mitochondrial stress-response. When the number of damaged mitochondria increases, TRB1 recruits ATG8 at the EMCS to regulate the formation of mitophagosomes. VAP27 may also recruit other unidentified autophagy proteins, such as ULK1/ATG1 and WIPI2/ATG18 to facilitate autophagosome maturation, as has been reported in mammalian cells. The model was created with BioRender.com.

It is known in yeast and mammalian systems that the ER-Mitochondrial interfaces are essential for autophagosomes biogenesis^3,16,39,41,49,50,57^, so it is also reasonable to propose that TRB1-VAP27 mediated ER-Mitochondria interaction and TRB1-ATG8 regulated mitophagy are two closely related processes. In animal cells, the homologues of VAP27 (VAPA/B) interact with multiple autophagy proteins, including ULK1/ATG1 and WIPI2/ATG18, to modulate autophagosome biogenesis^25^. In plants, VAP27 also functions in regulating actin-dependent autophagosome biogenesis through recruitment of AtEH1/Pan1^20^. Therefore, it is likely that the core autophagy machinery can also be recruited to the VAP27-mediated membrane interfaces, the VAP27-TRB1-mediated EMCSs, and function in selective autophagy processes (Fig. 8).

In the *trb1/trb2* mutant, mitochondrial structure and function are impaired, causing an overall reduction in ATP production and starch consumption (Fig. 4g–l). These phenotypes are indicative of defective mitochondrial function and are likely linked to the function of TRB1 in mitophagy and ER-Mitochondrial interaction, both of which are important in maintaining mitochondrial homeostasis and a healthy population of mitochondria through the turnover of damaged mitochondria. In animal cells, it has been observed that damaged sections of mitochondria become dissected from the entire mitochondrial network, and are degraded through mitophagy. Unsuccessful removal of damaged mitochondria causes overall mitochondria dysfunction and cell stress/death^10,12,47,52,53,58,59^. Consistent with this, *trb1/trb2*-loss-of-function results in the accumulation of damaged mitochondria under stress conditions caused by DNP treatment (Fig. 7d and Supplementary Fig. 10).

In conclusion, we have characterised a EMCS complex mediated by the interaction of VAP27 with the OMM-integral protein, TRB1, which has important roles in mitochondrial function through regulating ER-Mitochondrial interaction and mitophagy (Fig. 8a, b). Firstly, the TraB family proteins, TRB1 and TRB2 interact with ER-localized VAP27 to regulate ER-Mitochondrial interactions. Secondly, TRB1 interacts with ATG8 to regulate mitochondrial degradation working as putative mitophagy receptors, which is essential for normal plant growth in stressed conditions. We hypothesise that TRB1-mediated EMCSs are likely to regulate mitophagy, which in turn maintains healthy mitochondrial homeostasis and function. Whilst the mechanisms of EMCSs are already well known in animals and yeast, TRB1 is the identified protein to have a dual function in ER-mitochondrial interaction and mitophagy in plants, and these data aid in bridging the gap in our understanding of how EMCSs and mitophagy are regulated *in planta*.

## Methods

### Molecular cloning

The CDS of TRB1, TRB2, and TRB3 were amplified from a cDNA library generated using Arabidopsis seedling RNA using a RevertAid First Strand cDNA Synthesis Kit (Thermo Scientific, K1622), and then cloned into pMDC43 or pK7WGR2 via gateway cloning (BP Clonase™ II, Invitrogen 11789020; LR Clonase™ II, Invitrogen 11791020), to facilitate their expression as an N-terminal GFP or RFP fusion proteins. The CDS of ATG8e was amplified from the cDNA of ATG8e using Phanta Max Super-Fidelity DNA Polymerase (Vazyme, P505) and inserted into pK7WGR2 (N-terminal RFP fusion protein) and pK7WGC2 (N-terminal CFP fusion protein) via gateway cloning. To make the AIM motif mutation of TRB1, two predicted AIM motifs (Supplementary Fig. 4c) were mutated (F21A, I24A, and F279A, L282A) using their site-mutagenesis primers and a Mut Express II Fast Mutagenesis Kit-V2 (Vazyme, C214).

The mTAGBFP2-HDEL construct was generated as follows: A modified template for mTAGBFP2 was synthesised by Genscript, and PCR amplified with the primers HDEL-1 and HDEL-2 to include a c-terminal HDEL ER-retention signal, with a 3′ BsrGI restriction site. PCR primer extension of this coding sequence was performed using the primers HDEL-3, HDEL-4, and HDEL-5 to incorporate the coding sequence for an N-terminal WAK2 signal peptide^60^, and a 5′ SpeI restriction site. The resulting mTAGBFP2-HDEL sequence was inserted into the SpeI/BsrGI sites of pB7WG2 by restriction cloning using T7 DNA ligase (NEB). Mito-mTAGFP2 was generated as follows: mTAGBFP2 was PCR amplified with 5′ AscI and 3′ BstBI using 83 BFP-F and 83 BFP-R sites and cloned into the AscI/BstBI sites of pMDC83 using restriction cloning, to replace the GFP coding sequence with mTAGBFP2. The mitochondrial targeting signal^60^ was PCR amplified with 5′ SpeI and 3′AscI sites using the primers Mito-F and Mito-R and cloned into the SpeI/AscI site of pMDC83-mTAGBFP2, directly upstream of mTAGBFP2 to facilitate expression of Mito-mTAGBFP2.

For the expression of TRB1 and TRB2 under their own promoters, *pTRB1*:GFP-TRB1 and *pTRB2*:GFP-TRB2 native expression constructs were generated. Approximately 1500 bp of promoter region directly upstream of the TRB1 and TRB2 start codons was PCR amplified from Arabidopsis seedling genomic DNA with 5’ PstI and 3’ BamHI restriction sites using their gene-specific primers. The *pCAMV 35s*: promoters of pMDC43-TRB1 and pMDC43-TRB2 were excised by PstI/BamHI double restriction digest, and PstI/BamHI-digested *pTRB1* and *pTRB2* fragments were inserted respectively using T7 DNA Ligase (NEB, M0318S). For generation of recombinant TRB1-6xHIS protein, full length TRB1 CDS was PCR amplified from the cDNA of TRB1 with 5’ NdeI and 3’ NotI restriction sites using gene-specific primers, and subcloned into the NdeI/NotI restriction sites of pET24 using T7 DNA ligase (NEB, M0318S) to facilitate its expression as a C-terminal 6xHIS fusion protein in *E. coli*.

The FKBP-mCherry-TRB1^WT ΔTMD^ and FKBP-mCherry-TRB1^ΔAIMs ΔTMD^ constructs were expressed in pB7WG2 and were generated using hot fusion assembly of the following coding sequences, PCR amplified with homologous overlap of adjacent fragments. The template for the N-terminal FKBP-mCherry tag was acquired from VIB Ghent and PCR amplified using the primers FKBP-F and FKBP-R. TRB1^WT^ and TRB1^ΔAIMs^ were PCR amplified from the cDNA template using the primers TRB1 KSP-F and TRB1 KSP-R to generate coding sequences of TRB1^WT ΔTMD^ and TRB1^ΔAIMs ΔTMD^ lacking the C-terminal mitochondrial transmembrane domain (TMD) anchor. pB7WG2 was linearised by SpeI/BsrGI restriction digest to excise the gateway cassette, and FKBP-mCherry and TRB1^WT ΔTMD^ or TRB1^ΔAIMs ΔTMD^ were assembled into the linearised vector by hot fusion as previously described^61^ to generate FKBP-mCherry-TRB1^WT ΔTMD^ and FKBP-mCherry-TRB1^ΔAIMs ΔTMD^.

For yeast-two-hybrid constructs, full-length CDS of ATG8e in the intermediate vector was sub-cloned into pGADT7-GW plasmid using Gateway LR reaction. TRB1^WT^ and TRB1^ΔAIMs^ coding sequences without the transmembrane domains were amplified from intermediate vector and cloned into pGBKT7 plasmid by Gateway LR reaction. All primers used in this study were listed in Supplementary Table 1.

### Transgenic Arabidopsis lines

Mito-CFP, Mito-YFP, Mito-mCherry, GFP-HDEL organelle markers have been described previously^60^. To generate the GFP-ATG8a and RFP-TRB1 Arabidopsis lines, the GFP-ATG8a Arabidopsis line^20,62^ was dipped with RFP-TRB1 via Agrobacterium-mediated transformation. Arabidopsis lines co-expressing GFP-TRB1 + Mito-mCherry, GFP-TRB1 + VAP27-1-RFP, and GFP-TRB2 + Mito-mCherry were generated by crossing using stable transgenic plants of expressing single constructs. The GFP-TRB1/*atg5*, GFP-TRB1/*atg7* were generated by crossing of stable transgenic GFP-TRB1 plants and the *atg5* and *atg7* mutants. Other Arabidopsis mutants generated in this study and are described in detail below. Please refer to Supplementary Tables 2 and 3 for a complete list of all plasmid and transgenic Arabidopsis lines in this study.

### Arabidopsis Mutant analysis

The *trb1* (GABI KAT_369F08) T-DNA mutant line was obtained from GABI Kat, and the *trb2* (SALK_059433) T-DNA mutant line was obtained from NASC, and the *trb1*/*trb2*, and *vap27-1,3,4,6* CRISPR mutants were generated in this study. The *atg5* (SAIL_129_B07) and *atg7* (SAIL_11_H07) mutants have been described as previously^63^.

For T-DNA mutant lines, homozygosity was confirmed using the genotyping primers listed in Supplementary Table 1. Total RNA was extracted from seedlings (Qiagen) and cDNA was synthesised using Superscript III (Invitrogen). RT-PCR was used to confirm the absence of full-length transcripts in the T-DNA mutants using the primers listed in Supplementary Table 1. RT-PCR amplification of the housekeeping gene, *EF1ɑ*, was performed as a control, and equal amounts of Col-0 and mutant cDNA was used.

For CRISPR/Cas9-mediated generation of *trb1* and *trb2* mutants, two gRNA spacer sequences on the + strand were chosen as Cas9 targets: for TRB1; target 1 (5′-GCCGCCGCTAAACACAGAAT-3′) and target 2 (5′-GAAGACAGCTTGAAACAGTA-3′) were chosen. For TRB2; target 1 (5′- GGAGAAGTTGGAGCTGCCTG-3′) and target 2 (5′-ATTGCAGATAAAGGAACTGA-3′) were chosen. For *vap27-1/vap27-3* double mutants, two gRNA spacer sequences on the + strand of each gene were chosen as Cas9 targets. For VAP27-1, target 1 (5′-AGGTCTACTTGCGAAGTTCT-3′) and target 2 (5′-ATACTGGAGTTGTTCTCCCG-3′) were chosen; for VAP27-3, target 1 (5′-CGATAATTATGTCGCCTTCA-3′ and target 2 (5′-ATCCAAAGAAGTACTGCGTT-3′) were chosen. Target specificities were evaluated with CAS-OFFINDER, using an algorithm for potential off-target sites of Cas9 RNA-guided endonucleases^64^. The two respective TRB1 and TRB2 gRNA expression modules, were constructed via PCR using pCBC-DT1T2 as a template according to Xing et al.^65^ and Golden Gate assembled into the vector pHEE401E allowing egg cell-specific EC1.2en:EC1.1 promoter-controlled expression of 3× FLAG-NLS-zCas9-NLS^66^. The four VAP27-1/VAP27-3 gRNA expression modules were constructed via PCR using pCBC-DT1T2, pCBC-DT2T3 and pCBC-DT3T4 as a template according to Xing et al.^65^ and assembled via HiFi Gibson Assembly into pHEE401E. For *vap27-1,3,4,6* CRIPSR mutants, the two VAP27-4/VAP27-6 gRNA expression module was transformed into *vap27-1/vap27-3* CRISPR mutants generated above. The two gRNA expression module was constructed via Restriction-ligation cloning into pKSE401^66^, and the target (5′-TGGGACTCTTGCATCATTAG-3′) of VAP27-4, target (5′- GGACGAACACAGTATTTGCG-3′) of VAP27-6 were chosen respectively. Transformants were genotyped and sequenced using the primers listed in Supplementary Table 1.

### Generation of antibodies and immunostaining

Polyclonal antibodies were raised to full-length TRB1-6xHIS. TRB1-6xHIS was expressed in *E. coli* (Rosetta2; Novagen), and purified using Nickel-Agarose beads (Qiagen). Polyclonal antibodies were raised in mice as previously described^67^, the antigen was dialysed in PBS + 10% glycerol. Polyclonal antibodies were raised in 4–8 week old mice as previously described^67^. 100 µg antigen was used per boost, which were administered over a 2-month period on days 1, 14, 28, 42, and 56. The final antiserum was collected on day 63. Approval was granted by the project license holder and Animal Welfare and Ethical Review Board at Durham University.

For immunostaining, 5–7-day old seedlings were fixed in 4% PFA in PEM buffer (50 mM PIPES pH 6.9, 5 mM EGTA, 1 mM MgSO_4_) for 90 min. Fixed roots were washed (5 × 10 min) with PEM buffer and cell walls were digested using 0.5% cellulase, 0.05% pectolyase, 2% dricelase in PEM buffer + 0.4 M mannitol for 15 min. Roots were washed (3 × 10 min in PEM) and were gently squashed on a poly-L-lysine-coated coverslip. Roots were washed (3 × 10 min in PEM) and permeablised in PEM + 0.1% triton X100 for 15 min. Roots were washed (3 × 10 min in PEM) and incubated in blocking buffer 2% BSA in PEM) for 1 h. Samples were incubated in primary antibody at room temperature for one hour and then at 4 °C overnight. Samples were then washed in Phosphate Buffered Saline solution (PBS; 6 × 30 min) and were stained with secondary antibody at room temperature for one hour and then at 4 °C overnight. Samples were washed in PBS (6 × 30 min) and were mounted in Vectashield and imaged using Airy scanning super-resolution confocal microscopy.

For co-staining of TRB1 with VAP27-1 and mitochondria, Arabidopsis stable lines expressing native VAP27-1-RFP or Mito-mCherry were stained with anti-TRB1 mouse primary antibody at a dilution of 1:500 and anti-RFP rat primary antibody (Chromotek), followed by secondary antibody incubation with FITC conjugated against mouse, and TRITC conjugated against rat (Jackson ImmunoResearch).

### Live cell Imaging and Image analysis

*Nicotiana benthamiana* plants were grown in a growth room or greenhouse with long-day conditions. Transient expression was performed by leaf infiltration according to Sparkes et al.^68^. The transformed *N. benthamiana* tissues were imaged two days after infiltration using a laser scanning confocal microscope (Leica SP8). For each experiment, at least three independent infiltrations were performed. Images were taken in multi-track mode with line switching when multifluorescence was used. For GFP/RFP or FITC/TRITC combination, samples were excited at 488 and 552 nm and detected at 510–550 and 590–650 nm, respectively. For CFP/GFP/RFP, CFP was excited at 405 nm and detected at 450–480 nm; GFP was excited at 488 nm and detected at 550–580 nm; RFP was excited at 552 nm and detected at 590–650 nm. A Zeiss 800 laser scanning confocal microscope was used for mTAGBFP2/GFP/RFP and mTAGBFP2/YFP/mCherry imaging. mTAGBFP2 was excited at 405 nm and detected at 410–530 nm, GFP or YFP was excited at 488 nm and detected at 530–585 nm, and RFP and mCherry were excited at 561 nm and detected at 585–700 nm. Images were taken using a 63× oil immersion objective (NA = 1.4), with scan speed of 400 Hz at a resolution of 1024 × 1024 pixels. To capture the dynamics of TRB1, VAP27, mitochondria, and autophagosomes in Arabidopsis, cells from the root elongation zone and cotyledon were acquired at a fixed rate of 0.876 s per time point for 1 min. Airy scanning super-resolution confocal imaging was performed using the Zeiss LSM 880 confocal laser scanning microscope in fast AiryScan mode, and using a Zeiss C PL APO ×63 oil-immersion objective lens (NA = 1.4). Airy scanning confocal image raw data was processed using the Airyscan Processing tool on Zeiss Zen Black software.

FRET-FLIM was performed as previously described^17^. GFP-fusion proteins were used as fluorescence donor constructs, and RFP-fusion proteins were used as fluorescence acceptor constructs. As a negative control, the fluorescence lifetime of the GFP donor was measured when expressed alone, and compared to fluorescence lifetime of the donor co-expressed with an acceptor construct. TCSPC (time-correlated single photon counting) FRET-FLIM imaging of donor fluorescence lifetime was performed using a Leica SP5 CLSM fitted with a PicoQuant FLIM LSM upgrade kit and Leica SMD FLIM wizard software. PicoQuant Symphotime 32 software was used for data acquisition and analysis. GFP was excited using a 470 nm pulsed laser, and an internal photon counting detector with a 505–530 nm bandwidth was used to detect GFP fluorescence emission. Measurements were made from whole field images of cells expressing similar levels of donor and acceptor constructs, and continuous image acquisition was performed until 1000 photons were acquired at the brightest pixel. For each experimental series, 10 images were analysed.

For BiFC analysis, full-length CDS of TRB1 and VAP27-1 were sub-cloned into pMDC43-nYFP and pMDC43-cYFP plasmids, respectively. The plasmids were modified from a previous publication^69^, using a pMDC43 based vector for optimised expression level. Transiently transformed *N. benthamiana* were imaged two days after infiltration using a Leica SP8 confocal microscope. For nYFP/cYFP detection, samples were excited at 514 nm and detected at 550–580 nm; for nYFP/cYFP/CFP/RFP, images were excited at 405/514/552 nm and detected at 450–480/550–580/590–650 nm. For TMRM staining, seedlings were grown on MS medium for 5 days, and transferred into MS liquid medium with 500 nM TMRM for 10 min. Samples were excited at 552 nm and detected at 590–640 nm. Mitochondrial aspect ratio and fluorescence intensity was measured using Image J. For the images collected on the Zeiss microscopes, channel overlays were generated using the channel alignment tool in the Zeiss Zen Black software.

### Phylogenetic analysis

To identify TraB family homologues, the predicted proteins of each genome were searched using BLASTP with *Arabidopsis thaliana* TraB family proteins as input sequences. Used databases were NCBI (https://blast.ncbi.nlm.nih.gov/Blast.cgi), Phytozome (https://phytozome.jgi.doe.gov/pz/portal.html), and Orange Genome Annotation Project (http://citrus.hzau.edu.cn/cgi-bin/orange/search). Multiple alignments were constructed with the TraB domain amino acid sequence through SMART database (http://smart.embl-heidelberg.de). The TraB family tree was generated from multiple alignments by applying the Maximum Likelihood method based on the JTT matrix-based model^70^ to a bootstrapped dataset with 1000 replicates. Initial tree(s) for the heuristic search were obtained automatically by applying Neighbor-Join and BioNJ algorithms to a matrix of pairwise distances estimated using a JTT model, and then selecting the topology with superior log likelihood value. The tree is drawn to scale, with branch lengths measured in the number of substitutions per site. The analysis involved 44 amino acid sequences. All positions containing gaps and missing data were eliminated. There was a total of 74 positions in the final dataset. Evolutionary analyses were conducted in MEGA7^71^ v1.0.5877. See Supplementary Sequence data for a complete list of all protein sequences.

### Arabidopsis phenotype studies

For general growth phenotype studies, seedlings were grown on half MS medium for 7 days, and transferred to soil in a growth chamber (22 °C, 70% relative humidity, 14 h light/10 h dark cycle). Rosette perimeter and stem length measurement were performed according to previous studies^72^, seedlings were 30 and 40 days old, respectively. For DNP treatment and recovery, Arabidopsis seedlings were grown on MS medium for 5 days, and transferred onto medium supplemented with either DMSO or 50 μM DNP for 4 h, then the seedlings were transferred onto MS medium to recover for 2 days. Images were captured using a digital camera (Canon, EOS 80D), and the root length was measured using Image J.

### Yeast-two-Hybrid

Testing and control combinations of plasmids were co-transformed into yeast AH109 strain using the Frozen-EZ Yeast Transformation II Kit (ZYMO, T2001) and cultured on a SD/-Leu-Trp selective plate at 30 °C for 2–4 days. Three independent colonies of each combination of plasmids were picked and dotted on a SD/-Leu-His-Trp selective plate with 5 mM 3-AT. Combinations of pGBKT7-53 + pGADT7-T and pGBKT7-lam + pGADT7-T were used as positive and negative controls, respectively.

### Knocksideways in Plants (KSP)

Knocksideways in plants (KSP) was performed as described^44^. EB1a-mTAGBFP2-FRB was used as a rapamycin-dependent anchor for the FKBP-tagged bait and has been previously described^44^. The bait constructs FKBP-mCherry-TRB1^WT ΔTMD^ and FKBP-mCherry-TRB1^ΔAIMs ΔTMD^ were generated lacking the TRB1 mitochondrial transmembrane domain anchor to permit rapamycin-dependent delocalisation of the constructs to the EB1a-mTAGBFP2-FRB anchor. YFP-ATG8e was used as the prey construct. *N. benthamiana* plants transiently expressing combinations of either EB1a-mTAGBFP2-FRB, YFP-ATG8e and FKBP-mCherry-TRB1^WT ΔTMD^, or EB1a-mTAGBFP2-FRB, YFP-ATG8e FKBP-mCherry-TRB1^ΔAIMs ΔTMD^ were infiltrated with 1 µM Rapamycin in MQ water (diluted from a 1 mM stock in DMSO) for 1 h before imaging.

### Proteomics screen, co-immunoprecipitation, and western blotting

The TAP-tag purification and mass spectrometry was performed by a proteomic service performed in Ghent University, as described previously^73,74^. Cloning of transgenes encoding GS^rhino^ tag^74^ fusions under control of the constitutive cauliflower tobacco mosaic virus 35S promoter and transformation of Arabidopsis cell suspension cultures (PSB-D) with direct selection in liquid medium were carried out as previously described^73^. TAP experiments were performed with 100 mg of total protein extract as input as described in Van Leene et al., 2015 with minor modifications. Briefly, for protein extraction prior to the affinity purification, the detergent Nonidet P-40 was replaced by digitonin or Triton-X-100. Crude protein extracts were prepared in extraction buffer without detergent. After the mixing step, digitonin or Triton-X-100 was added to a final concentration of 1% (w/v) and extracts were incubated for 1 h at 4 °C under gentle rotation. A soluble protein fraction was obtained by centrifugation at 36,900 × *g* for two times 20 min at 4 °C. In all further steps, the detergent 0.1% (v/v) Nonidet P-40 was replaced by 0.2% (w/v) digitonin or Triton-X-100. Protein interactors were identified by LC-MS/MS using an LTQ Orbitrap Velos (ThermoFisher Scientific) as described in Van Leene et al., 2015. Proteins with at least two matched high confident peptides were retained. Background proteins were filtered out based on frequency of occurrence of the co-purified proteins in a large dataset containing 543 TAP experiments using 115 different baits^74^. The mass spectrometry proteomics data have been deposited to the ProteomeXchange Consortium via the PRIDE^75^ partner repository with the dataset identifier PXD036285 and 10.6019/PXD036285.

The immunoprecipitation assays were performed using GFP-Trap_A (Chromotek, gtma-20) as described previously^20^. About 0.2 g of plant material was ground in liquid nitrogen and resuspended in lysis buffer containing 10 mM Tris-HCl, pH 7.5, 150 mM NaCl, 0.5 mM EDTA, 1 mM phenylmethylsulfonyl fluoride, 0.5% Triton-X100 and Complete Protease Inhibitor (Sigma). The mixture was incubated on ice for 2 h and then centrifuged at 2500 × *g* for 2 min at 4 °C, the supernatant and agarose pellet were harvested in separate tubes for analysis. For detection, the membrane was incubated in 2 × TBST buffer with 5% milk prior to primary antibody incubation (1:1000 for anti-RFP, Abcam ab62341; 1:2000 for anti-GFP, Biorbyt orb323045) at room temperature for 3 h. After three washes in TBST buffer, the membrane was probed with HRP conjugated mouse secondary antibody (Yeasen, 33201ES60) at 1:5000 and developed using a Super ECL reagent (Yeasen, 36208ES60).

### Electron microscopy

All samples were prefixed in 2.5% glutaraldehyde (v/v in 0.1 M phosphate buffer, pH 7.2) for 2 h, and then rinsed 3 times with 0.1 M phosphate buffer (pH 7.2). They were post-fixed in 1% OsO_4_ for 2 h, followed by three 15 min rinses with phosphate buffer. Afterwards, the samples were dehydrated through an acetone series (30, 50, 70, 90, 100, 100, and 100%) (v/v in dd H_2_O) at room temperature, samples were incubated for 20 min at each concentration. Then the samples were infiltrated in a graded scale of 3:1, 1:1, 1:3 (v/v) acetone/SPI-PON 812 resin and, as the last step, in 100% (v/v) SPI-PON 812 resin (SPI Supplies, West Chester), for 12 h per step. Samples were embedded in SPI-PON 812 resin and polymerized at 60 °C for 48 h. Ultrathin sections (80 nm) were prepared using an EM UC7 Ultracut ultramicrotome (Leica, UC7). Sections were observed and photographed using a transmission electron microscope (Hitachi H-7650) at an accelerating voltage of 80.0 kV. For ER-mitochondria quantification, the shortest distance between mitochondria and associated ER membrane was measured and at least 20 mitochondria from three independent biological samples were analysed.

### Drug treatment and autophagy assays

For Conc A treatment, Arabidopsis seedlings were treated with either DMSO or 1 μM Conc A for 8–12 h prior to imaging. For the Conc A & DNP treatment, Arabidopsis seedlings were first grown on MS medium for 5 days, and transferred into liquid MS medium supplemented with Conc A for 12 h, then DNP was added into the same medium to a concentration of 50 μM, the seedlings were incubated for an additional 2 h prior to microscopy.

For autophagy-flux tests, seedlings were treated with 1 μM Conc A before western blotting, the dilution ratio of primary antibody was 1:2000 for anti-GFP (Biorbyt orb323045) and HRP conjugated mouse secondary antibody (Yeasen, 33201ES60) was 1:5000.

### Real-time qPCR

RNA from various samples (e.g., roots, stems, leaves, flowers) were extracted according to the instructions of the Universal Plant RNA Isolation Kit (NOBELAB, RNE35). The concentration of RNA was measured by NANODROP 2000 ultraviolet. 1 µg RNA was used to synthesize the first-strand cDNA by HiScript II Q RT SuperMix (Vazyme, R223). Finally, the gene expression of *AtTRB1* and *AtTRB2* were detected in a qPCR system (Roche, Lightcycler 480). ChamQ Universal SYBR qPCR Master Mix (Vazyme, Q711) was used to perform the reaction. The relative expression level of each gene was quantified with the comparative threshold cycle method, using actin as the internal reference. Reactions for each of the three biological replicates were performed in duplicate. The primer sequences used in RT-qPCR are shown in Supplementary Table 1.

### Starch Assay

Rosettes of four-five weeks old plants were collected and immediately transferred to 95% ethanol, and boiled until completely decoloured (about 10–15 min). Samples were stained in 5% Lugol’s solution (5% [w/v] I_2_ and 10% [w/v] KI) for 10 min, and then destained in water until a clear background was obtained before imaging^76^. For starch content measurement, lugol-stained samples were washed using deionised water until decoloured, air dried at room temperature and ground into powder. The powdered tissue was washed three times with 2 ml of diethyl ether and subsequently washed twice with 80% ethanol, and then twice with ddH_2_O. The washed powder was boiled in 5 mL ddH_2_O for 15 min. 980 μL of the supernatant was mixed with 20 μL of staining solution [5% (w/v) I_2_ and 2% (w/v) KI]. This reaction was subjected to colorimetric determination at 660 nm. The standard curve was performed with standard starch (Sigma, 33615).

### ATP measurement

ATP, ADP, and AMP content was measured using HPLC. Sample preparations and adenylate standards for the measurement were performed as previously reported^77^. Briefly, 0.1–0.2 g of Arabidopsis leaves were grinded in liquid nitrogen and transferred to a centrifuge tube, 500 μl of 0.1 M HCl was added and vortexed two times for 15 s on ice. The mixture was centrifuged at 15,000 × *g* for 10 min at 4 °C. 15 μl of the supernatant mixed with 77 μl Citric acid-phosphate buffer^77^ and 8 μl 45% chloroacetaldehyde and incubated at 80 °C for 10 min. The mixture was centrifuged at 12,000 × *g* for 30 min at room temperature. The supernatant was filtered through a 0.22 μm microporous membrane and loaded into a HPLC. The analysis of adenosines was performed by HPLC on a C_18_ column (Acclaim® PolarAdvantage II, 4.6 × 150 mm, 3 μm, 120 Å) connected to an HPLC system (1525 binary HPLC pump coupled with 2998 photodiode array detector and 2707 autosampler, Waters). The HPLC analysis was carried out as described previously^78^. The gradient for separation of adenosine derivatives was optimized as follows: 0 min, 100% A; 2 min, 95% A and 5% B; 6 min, 80% A and 20% B; 7 min, 75% A and 25% B; 8–10 min, 100% A (running buffer A: 0.06 M K_2_HPO_4_, 0.04 M KH_2_PO_4_, pH 7.0; running buffer B: 100% Acetonitrile). The flowrate is set to 1.2 ml/min.

### Statistical analysis

All statistical graphs were performed using the GraphPad Prism software (ver. 7.00). The results were compared using appropriate statistical analysis methods (Student’s t tests, one-way ANOVA or two-way ANOVA for multiple comparisons) and data were expressed as the mean ± SEM (Std. Error of Mean) with each symbol. *P* value below 0.05 was considered significant.

### Accession numbers

The Arabidopsis Genome Initiative locus identifiers for the genes mentioned in this article are TRB1 (AT1G05270), TRB2 (AT2G32340), TRB3 (AT5G52030), VAP27-1 (AT3G60600), VAP27-3 (AT2G45140), VAP27-4 (AT5G47180), VAP27-6 (AT4G00170), ATG8a (AT4G21980), ATG8e (AT2G45170), ATG5 (AT5G17290), and ATG7 (AT5G45900).

### Reporting summary

Further information on research design is available in the Nature Research Reporting Summary linked to this article.

## Supplementary information

Supplementary Information

Reporting Summary

## Data Availability

The authors declare that all data supporting the findings of this study are available within the article and its Supplementary Information files, or from the corresponding author upon reasonable request. The mass spectrometry proteomics data are available via ProteomeXchange with identifier PXD036285. Source data are provided with this paper.

## Source data

Source Data
