# Supplementary material for: TraB family proteins are components of ER-mitochondrial contact sites and regulate ER-mitochondrial interactions and mitophagy
Source: Nat Commun. 2022 Sep 26;13:5658. doi: 10.1038/s41467-022-33402-w (PMC9513094; doi:10.1038/s41467-022-33402-w)
Supplement: Supplementary file 1 — Supplementary Information [file 41467_2022_33402_MOESM1_ESM.pdf]

TraB family proteins are component of the ER-Mitochondrial contact site regulating ER-Mitochondrial interaction and mitophagy

Li and Duckney et al., Supplementary information

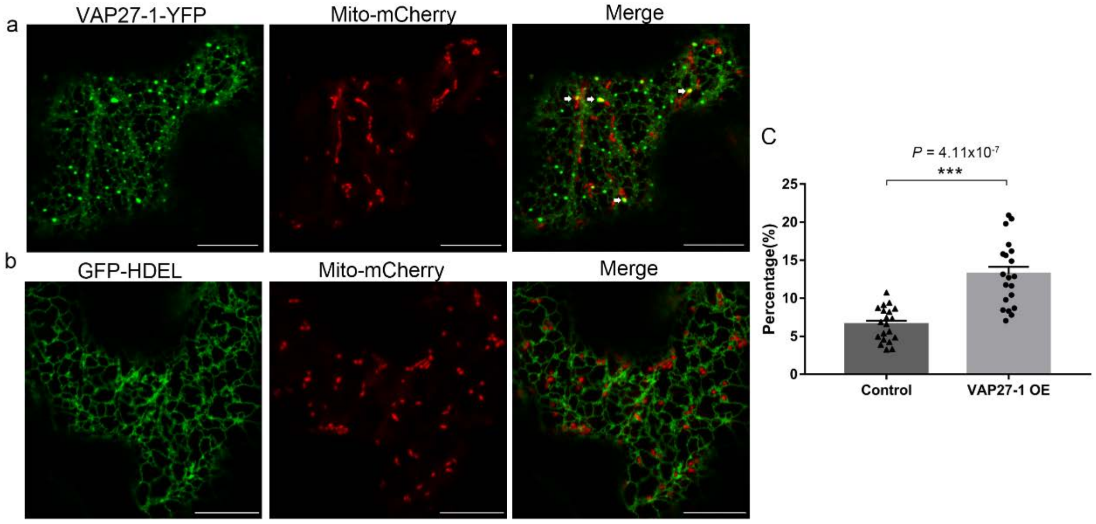

Supplementary Figure 1. a-b. Mitochondria co-expressed with VAP27-1-YFP (a) and GFP-HDEL (b) in *N. benthamiana* leaf epidermal cells; partial co-localization between VAP27-1 and mitochondria at the ER-mitochondrial interface is identified (arrow). **c.** Quantifications of mitochondria over-lapped with the ER in the presence of VAP27-1. n = 20 cells examined over 3 independent experiments, error bars are SEM, *** $P < 0.001$ in two-tailed Student's t tests (Scale bar = 10 μm).

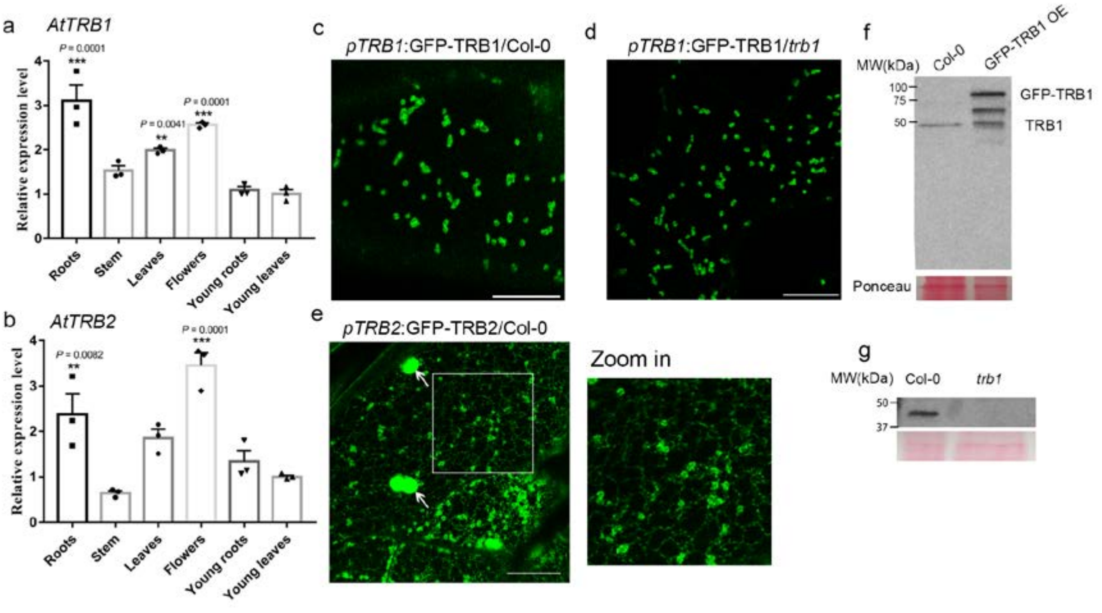

Supplementary Figure 2. a-b. RT-PCR result demonstrated that both TRB1 and TRB2 are ubiquitously expressed. **c-e.** Their subcellular localization in leaf tissues is confirmed using GFP fusions driven by their endogenous promoters, in either wild type (c, e) or *trb1* mutant background (d). Please note the bright structures (arrow) are chloroplasts that produce strong auto-fluorescence. **f.** Western blot analysis using the TRB1 antibody; the wild type and 35S:GFP-TRB1 transgenic Arabidopsis extracts were used and both endogenous TRB1 and over-expressed GFP-TRB1 were detected. **g.** Western blot analysis of TRB1 antibody. The antibody detects a band in an Arabidopsis root protein extract corresponding to the size of TRB1, but does not detect a similar band in the *trb1* CRISPR mutant root tissue. Microscopic imaging were repeated at least 3 times with similar results and western blots were repeated two times with similar results. n = 3 independent experiments for qRT-PCR, error bars are SEM, ** 0.001 < P < 0.01, *** P < 0.001 in one-way ANOVA, Dunnett's multiple comparisons test (Scale bar = 10 μm).

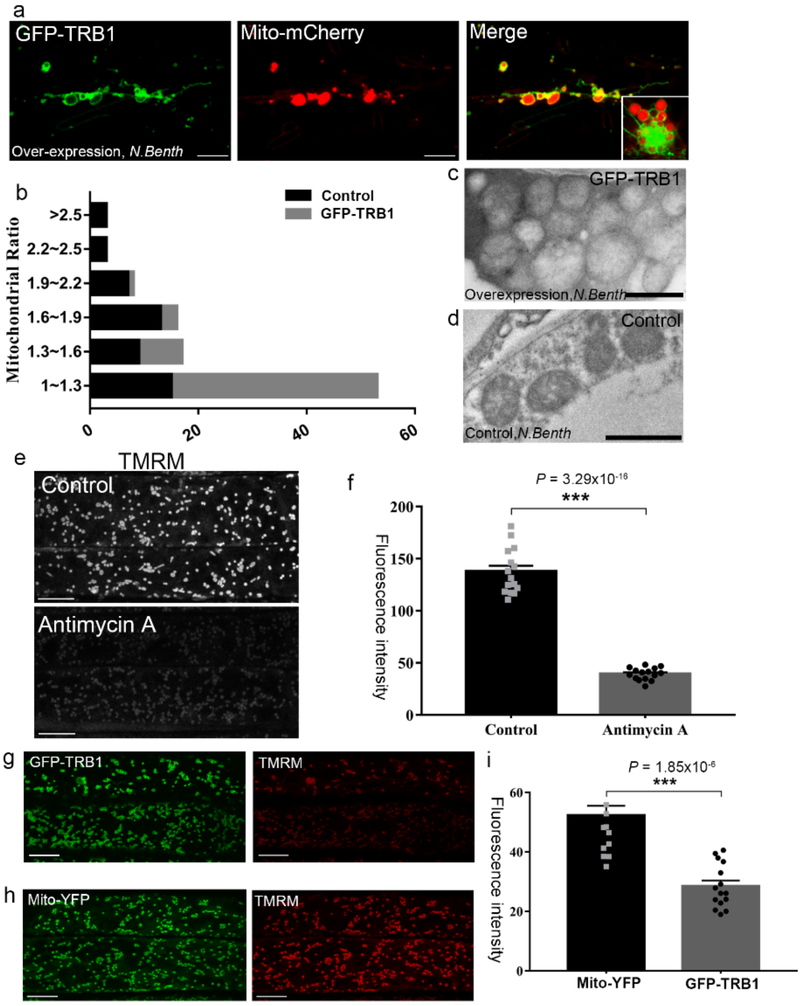

Supplementary Figure 3. TRB1 over-expression affects mitochondrial function, as indicted by morphological studies and TMRM labelling. **a.** In *N. benthamiana* leaf epidermal cells, over-expressing GFP-TRB1 induces mitochondrial enlargement, swelling and aggregation. **b.** Mitochondria became more circular in the presence of over-expressed TRB1, as indicated by the length-width ratio. **c-d.** TEM study of *N. benthamiana* leaf epidermal cells in the presence/absence of over-expressed GFP-TRB1 (as in a). The mitochondria are more aggregated and electron transparent at the ultrastructural level. **e.** Antimycin treatment reduces mitochondrial electron potential, as indicated by the reduction of fluorescent intensity of TMRM stained mitochondria. **f-g.** In the presence of GFP-TRB1, mitochondrial electron potential (TMRM stained) is reduced significantly. n = 50 mitochondrial numbers for mitochondrial ratio analysis; n = 15 cells examined over 3 independent experiments for confocal quantification, error bars are SEM, *** $P < 0.001$ in two-tailed Student's t tests and with Welch's correction in **i** (Scale bar = 10 µm for confocal; Scale bar = 500 nm for TEM)

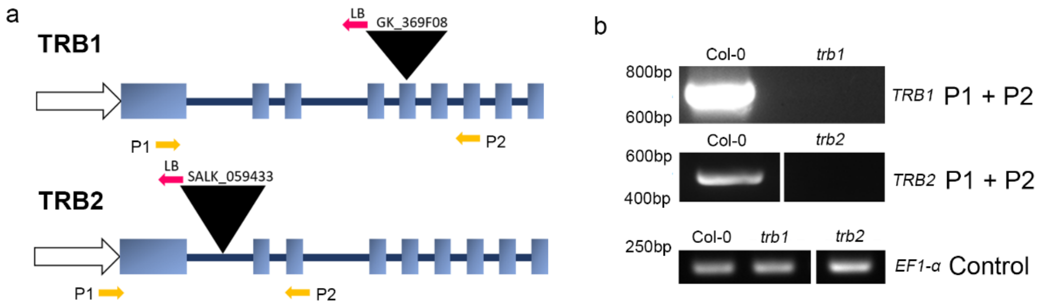

Supplementary Figure 4. a. Diagrammatic illustration of the T-DNA mutant of *trb1* and *trb2* used in this study. **b.** RT-PCR analysis of *trb1* and *trb2* T-DNA mutant lines using primers listed in Supplementary Table 1. EF-1α was used as the loading control in Col-0, *trb1* and *trb2* mutants demonstrating that equal quantities of cDNA were used for RT-PCR analysis. Separate panels represent amplified transcripts from the same experiment. RT-PCRs were repeated at least three times with similar results.

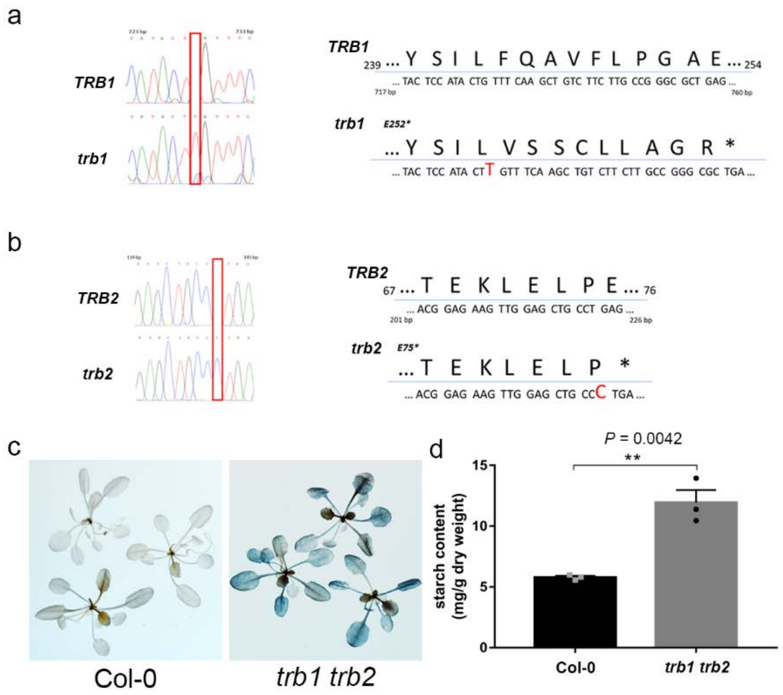

Supplementary Figure 5. a-b. Diagrammatic illustration of the CRISPR mutant of *trb1/trb2* generated in this study; the site of mutation is highlighted in red. **c-d.** The starch levels in Col-0*, trb1/trb2* CRISPR plants (4 weeks old) were analysed by Lugol staining; the mutant accumulated a high level of starch by the end of the light cycle (d). Such results were confirmed by spectrophotometry (d). n = 9 biologically independent plants examined over 3 independent experiments for starch quantification, error bars are SEM, ** $0.001 < P < 0.01$ in two-tailed Student’s t test.

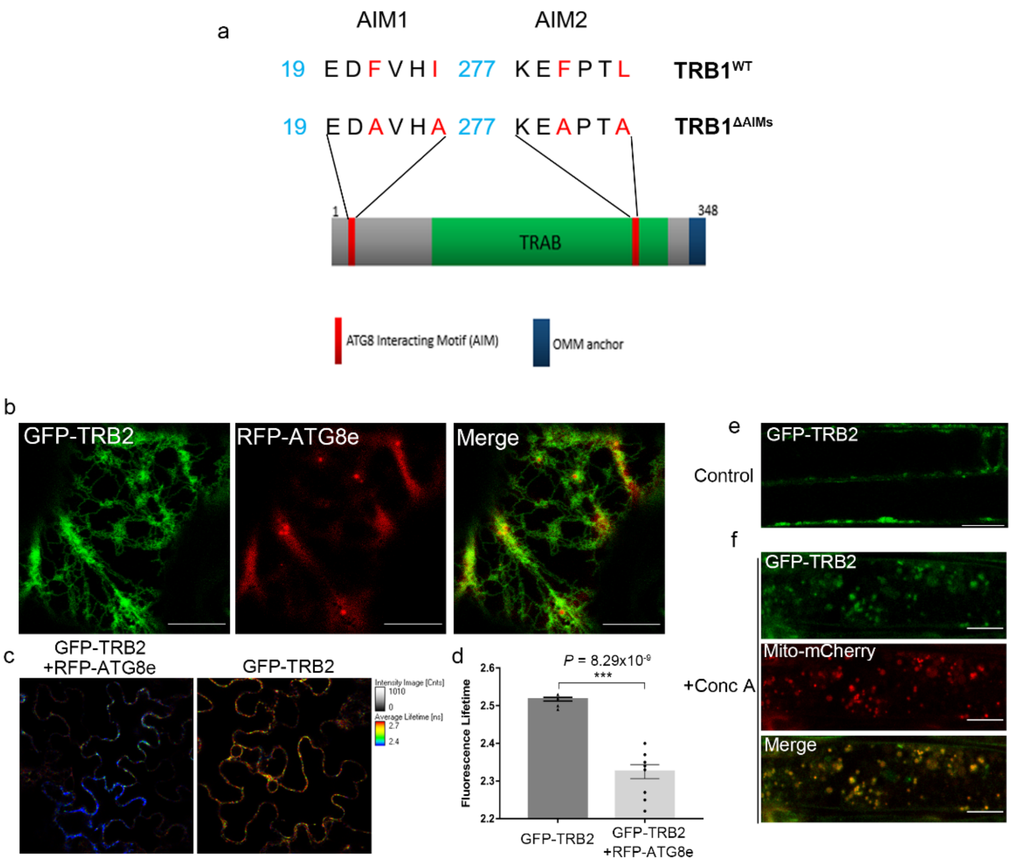

Supplementary Figure 6. a. Diagrammatic illustration of the sequence of two ATG8-interaction motifs within TRB1. **b.** GFP-TRB2 and RFP-ATG8e exhibit partial co-localization in *N. benthamiana* leaf epidermal cells under non-stressed condition. **c-d.** FRET-FLIM demonstrate the interactions between TRB2 and ATG8. The fluorescence life-time of GFP-TRB2 (control) changes significantly (life-time reduces from 2.52 ± 0.01 ns to 2.33 ± 0.02 ns) in the presence of RFP-ATG8e, indicating a physical interaction. **e-f.** Transgenic Arabidopsis plants expressing GFP-TRB2 or Mito-mCherry + GFP-TRB2 grown in MS growth media without stress. After Conc A treatment, numerous mitochondria (co-labelled with Mito-mCherry + GFP-TRB2) accumulate in the vacuole. Microscopic studies were repeated at least 3 times **(b)** or 2 times **(e-f)** with similar results. n = 10 biologically independent samples for FRET-FLIM analysis, error bars are SEM, *** $P < 0.001$ in Mann Whitney test (Scale bar = 10 µm).

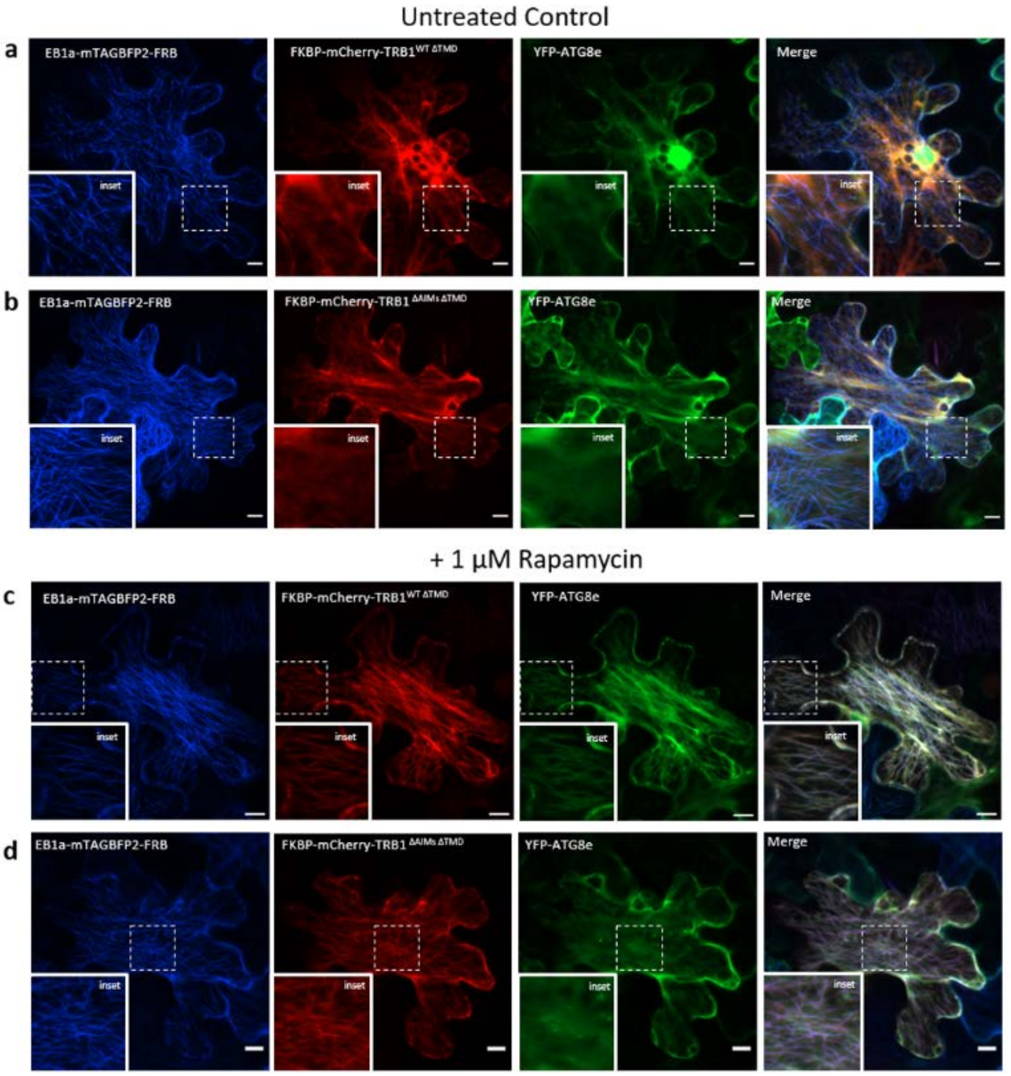

Supplementary Figure 7. TRB1 interacts with ATG8 in the knocksideways in plants (KSP) system. Combinations of either EB1a-mTAGBFP2-FRB, YFP-ATG8e and FKBP-mCherry-TRB1$^{WT\ \Delta TMD}$ (a, c) or EB1a-mTAGBFP2-FRB, YFP-ATG8e and FKBP-mCherry-TRB1$^{\Delta AIMs\ \Delta TMD}$ (b, d) were transiently expressed in *N. benthamiana.* (a, b): Prior to rapamycin treatment, FKBP-mCherry-TRB1$^{WT\ \Delta TMD}$, FKBP-mCherry-TRB1$^{\Delta AIMs\ \Delta TMD}$ and YFP-ATG8e did not colocalise with EB1a-mTAGBFP2-FRB. (c, d): Following treatment with rapamycin, both FKBP-mCherry-TRB1$^{WT\ \Delta TMD}$ and FKBP-mCherry-TRB1$^{\Delta AIMs\ \Delta TMD}$ were delocalised to microtubule cytoskeleton to colocalise with EB1a-mTAGBFP2-FRB. (c): When co-expressed with FKBP-mCherry-TRB1$^{WT\ \Delta TMD}$, YFP-ATG8e was also pulled from the cytosol to the microtubule cytoskeleton after rapamycin treatment, and was observed to colocalise with FKBP-mCherry-TRB1$^{WT\ \Delta TMD}$, consistent with interaction between TRB1^{WT} and ATG8e. (d): When co-expressed with FKBP-mCherry-TRB1$^{\Delta AIMs\ \Delta TMD}$, YFP-ATG8e was not pulled to the microtubule cytoskeleton, and did not colocalise with mCherry-TRB1$^{\Delta AIM\ \Delta TMD}$. This indicates no interaction between YFP-ATG8e and mCherry-TRB1$^{\Delta AIMs\ \Delta TMD}$. These findings were consistently observed and ≥ 10 cells were analysed per experimental series. Scale Bars = 10 µm.

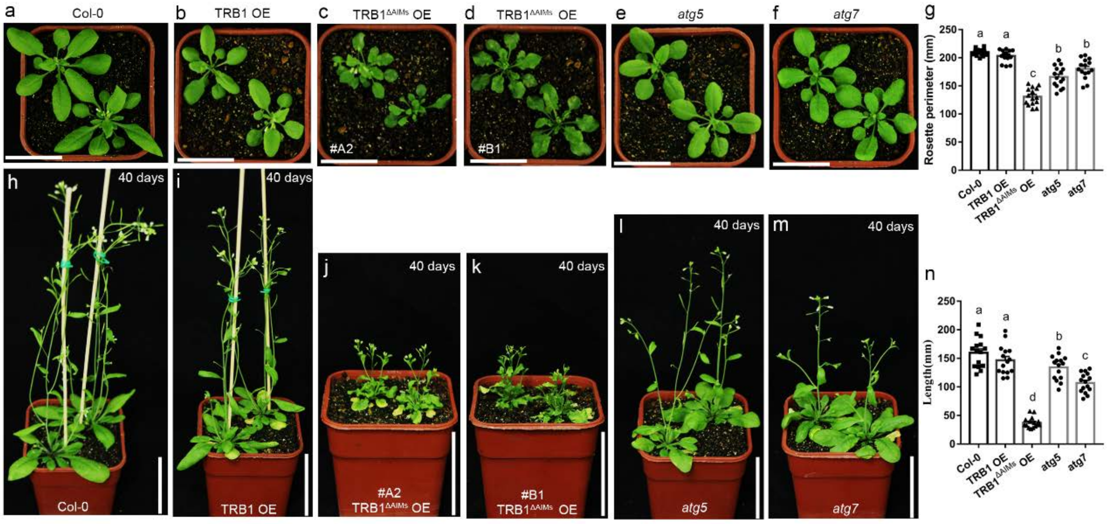

Supplementary Figure 8. The over-expression of the TRB1 mutant that is defective in ATG8 interaction (TRB1$^{\Delta AIMs}$) exhibits severe developmental defects. **a-f.** Arabidopsis of Col-0 (a), TRB1 over-expressing line (b), TRB1$^{\Delta AIMs}$ over-expressing lines (c-d), *atg5* (e), atg7 (f) at 30 days after germination. **g.** Plant development and the size of rosette leaves (rosette perimeter) were quantified. **h-m.** Arabidopsis Col-0 (h), TRB1 over-expression line (i), TRB1$^{\Delta AIMs}$ over-expression lines (j-k), *atg5* (l), *atg7* (m) at 40 days after germination. **n.** The stem length of plants was quantified. n = 15 biologically independent plants for measurement, error bars are SEM, different letters indicate significant differences in one-way ANOVA ($P < 0.05$), Tukey's multiple comparisons test. Scale bar = 5 cm (a-f); Scale bar = 10 cm (g-l).

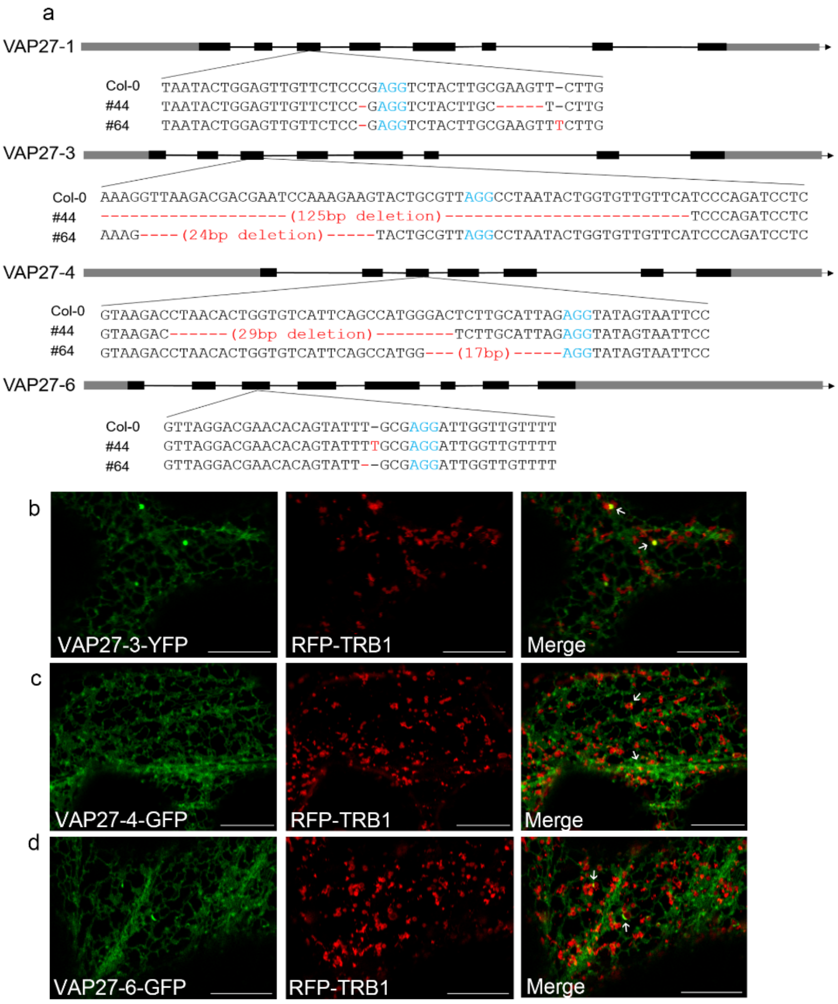

Supplementary Figure 9. a. Diagrammatic illustration of the CRISPR mutant of the *vap27-1,3,4,6* mutant generated in this study; the site of mutation is highlighted in red. **b-d.** Transient co-expression of RFP-TRB1 with different VAP27 homologues in *N. benthamiana* leaf epidermal cells. Microscopic imaging were repeated at least 3 times with similar results that the ER localized VAP27 (VAP27-3, VAP27-4 and VAP27-6) exhibit partial co-localization (arrow) with TRB1 at putative ERMCs (Scale bar = 10 µm).

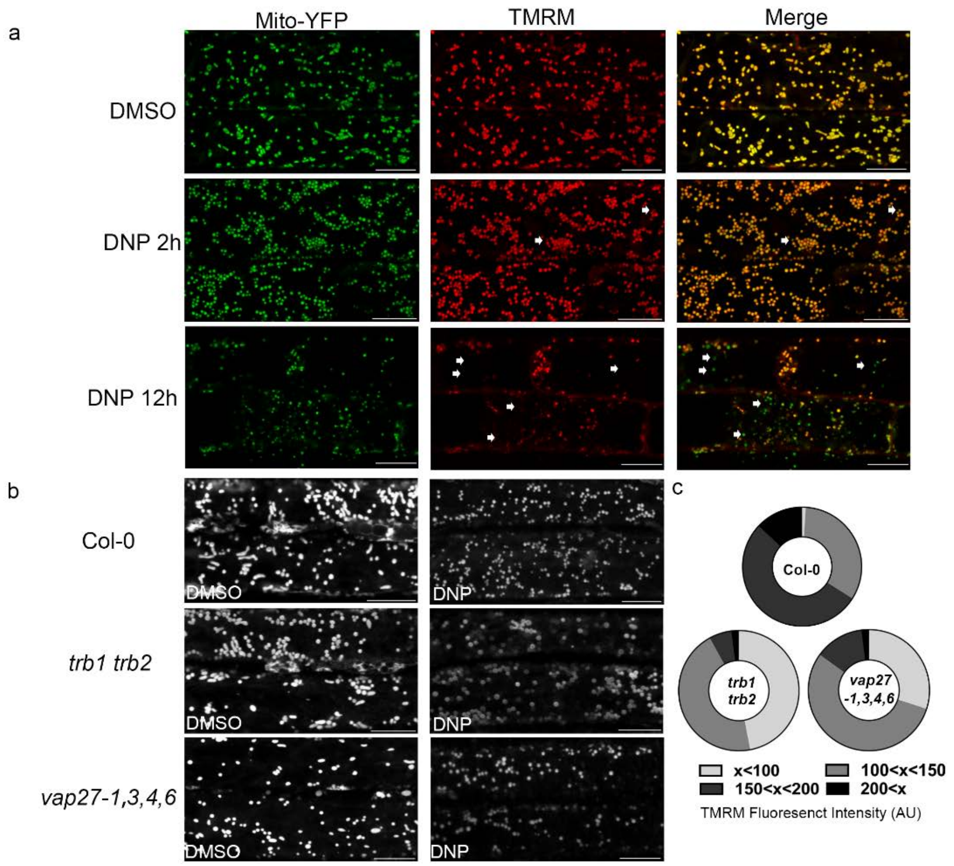

Supplementary Figure 10. The accumulation of impaired mitochondria after DNP treatment in the *trb1/trb2* and the *vap27-1,3,4,6* mutant. **a.** Transgenic Arabidopsis expressing Mito-YFP was treated with DNP to induce mitochondrial depolarization; after 2 hours of treatment, a few mitochondria are weakly labelled by TMRM (arrow); after prolonged treatment (12h), the majority of mitochondria have lost their membrane potential and cannot be stained by TMRM (arrows pointing to the mitochondria that are Mito-YFP positive but TMRM negative). **b-c.** Arabidopsis (Col-0, *trb1/trb2* and *vap27-1,3,4,6*) were treated with DNP for 2 hours and their mitochondrial membrane potential was labelled by TMRM. In both the *trb1/trb2* and the *vap27-1,3,4,6* mutant, the proportion of mitochondria with reduced TMRM labelling is significantly increased (c), suggesting that the large population of mitochondria that accumulated in the cytoplasm are dysfunctional in these mutants. n = 100 mitochondrial numbers examined over 20 cells for mitochondrial membrane potential analysis (Scale bar = 10 µm).

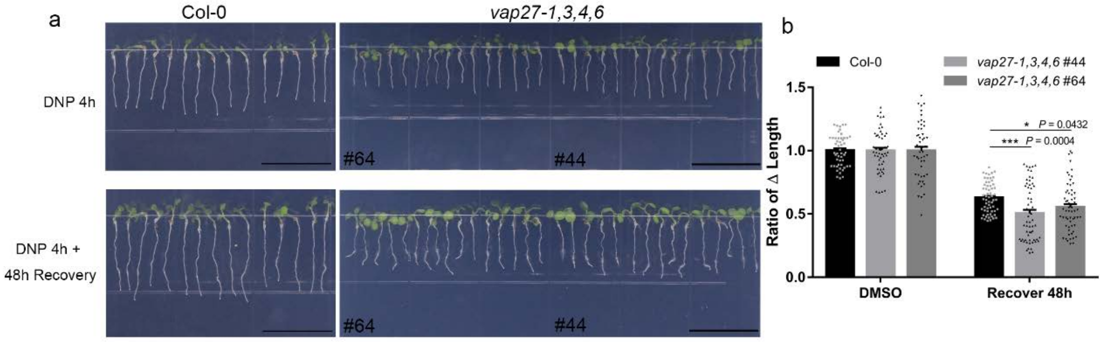

Supplementary Figure 11. a-b. The recovery of *vap27-1,3,4,6* mutant seedlings (two independent lines) after 4 hours of DNP treatment was studied. The root growth of the *vap27-1,3,4,6* mutant plants is significantly reduced compared to that in the wild type, suggesting that the DNP induced mitochondrial dysfunction has more impact on plant development when the function of TRB is impaired. Root length after recovery was normalised against non-treated plants (b). n = 60 biologically independent plants for root analysis under DNP treatment, error bars are SEM, * 0.01 < *P* < 0.05, *** *P* < 0.001 in two-way ANOVA, Dunnett's multiple comparisons test (Scale bar = 1.5 cm).

Supplementary Table 1 The list of primers used in this study

Purpose		Name	Sequence (5’→3’)
Gateway clone	TRB1	TRB1 Gat F	GGGGACAAGTTTGTACAAAAAAGCAGGCTGCATGACGATAGAGCCGACGC
		TRB1 Gat R	GGGGACCACTTTGTACAAGAAAGCTGGGTTCACTTTCTTCTTGAAAGAAGTATACCCG
	TRB2	TRB2 Gat F	GGGGACAAGTTTGTACAAAAAAGCAGGCTTCACCATGGAGTTGAAGCCGGAGGTTC
		TRB2 Gat R	GGGGACCACTTTGTACAAGAAAGCTGGGTCTTACTTAGCAAGATACATTCCGG
	TRB3	TRB3 Gat F	GGGGACAAGTTTGTACAAAAAAGCAGGCTGCATGAAACTCCTTCCGAATCCAT
		TRB3 Gat R	GGGGACCACTTTGTACAAGAAAGCTGGGTTCATGAAAGGTTTTGATTCATCATCAG
	ATG8e	ATG8e Gat F	GGGGACAAGTTTGTACAAAAAAGCAGGCTCCATGAATAAAGGAAGCATCTTTAAG
		ATG8e Gat R	GGGGACCACTTTGTACAAGAAAGCTGGGTTTAGATTGAAGAAGCACCGAATG
	TRB1 without TMD	TRB1 TM F	GGGGACAAGTTTGTACAAAAAAGCAGGCTGCATGACGATAGAGCCGACGC
		TRB1 TM R	GGGGACCACTTTGTACAAGAAAGCTGGGTTCATCTCTTTAACGTAAACACTGAC
	Citrus TRB1	CsTRB1 Gat F	GGGGACAAGTTTGTACAAAAAAGCAGGCTGCATGAATCGCTTGTCTCGTCTAG
		CsTRB1 Gat R	GGGGACCACTTTGTACAAGAAAGCTGGGTGTTACTTTTTGCAGTTAATATAGATG
TRB1 ΔAIMs mutant primer	F21A, I24A	ΔAIM1 F	GACGCCGTTCACGCCGATGACCCTAGGCCTACCGGCGATA
		ΔAIM1 R	ATCGGCGTGAACGGCGTCCTCTCCAGAGTGAACCTCCGGC
	F279A, L282A	ΔAIM2 F	GAAGCTCCAACTGCCATGGATACAATTGTGCATGAGCG
		ΔAIM2 R	CATGGCAGTTGGAGCTTCCTTGCTCATTTCTTGAATC
Ligation of TRB promoter	*pTRB1* into pMDC43	*pTRB1* PstI F	GAACTGCAGCAACACCTCCTCAGAAGAAG
		pTRB1 BamHI R	GAAGGATCCCGAAATCGAGGAGATGATGGC
	pTRB2 into pMDC43	*pTRB2* PstI F	CCCCTGCAGTAGTATCCTTCTTCACATCGAC
		pTRB2 BamHI R	GAGGATCCTACGCGAGGAGTGATTGGCG
Ligation of TRB1 CDS into pET24		TRB1 NdeI F	GGGCATATGATGACGATAGAGCCGACGCA
		TRB1 NotI R	GGGGCGGCCGCCTTTCTTCTTGAAAGAAGTATACC
Genotyping of T-DNA insert mutants	*trb1* (GAB_I369F08)	*trb1* F	CACAAAGCAGAGGCTGCCTATATCTTAGAG
		trb1 R	CCATGAGAGTTGGAAATTCCTTGCT
	trb2 (SALK_059433)	*trb2* F	GCGTTACCTCCTTCGACGATCAGAC
		trb2 R	CGACTTGAACACAACTCCAAAAAGACAG
	atg5 (SAIL_129_B07)	*atg5* LP	ATTTGCTATTTGTTTGGCACG
		atg5 RP	TACCGTTCATGACAGAGGTCC
	atg7 (SAIL_11_H07)	*atg7* LP	CAGCGTGATCTGTGAGAACTG
		atg7 RP	TTCTTGGAGCTGGTACATTGG
	LB of SAIL	LB1	GCTTTTCAGAAATGGATAAATAGCCTTGCTTCC
	LB of SAIK	LBb1.3	ATTTTGCCGATTTCGGAAC
	LB of GABI	o8760	GGGCTACACTGAATTGGTAGCTC
RT-PCR analysis	*trb1* (GAB_I369F08)	*trb1* RT-F	GGAGGAAGAATTTAGAGGCTCCG
		trb2 RT-R	GGTCTCGCTCATGCACAATTGTATCC
	trb2 (SALK_059433)	*trb2* RT-F	GCGTTACCTCCTTCGACGATCAGAC
		trb2 RT-R	CGACTTGAACACAACTCCAAAAAGACAG
	loading control	*EF1α* F	CCCATTTGTGCCAATCTCT
		EF1α R	CACCGTTCCAATACCACCAA

RT-qPCR analysis	*TRB1*	*TRB1* qF	TCACATCGATGACCCTAGGC
		TRB1 qR	AGTAGCCTCCGATGAACACT
	TRB2	*TRB2* qF	ACCAACAGCTTAGTGAATGTGG
		TRB2 qR	CCGCCACACACTCAACATAA
	Internal reference	*ACTIN2* qF	TGGCGATGAAGCTCAATCC
		ACTIN2 qR	CACTGGCATAAAGAGAAAG
Golden gate cloning	TRB1 gDNA into pHEE401E	TRB1 DT1 F	ATATATGGTCTCGATTGCCGCCGCTAAACACAGAATGTTTTAGAGCTAGAAATAGC
		TRB1 DT2 R	ATTATTGGTCTCGAAACTACTGTTTCAAGCTGTCTTCAATCTCTTAGTCGACTCTAC
	TRB2 gDNA into pHEE401E	TRB2 DT1 F	ATATATGGTCTCGATTGGAGAAGTTGGAGCTGCCTGGTTTTAGAGCTAGAAATAGC
		TRB2 DT2 R	ATTATTGGTCTCGAAACTCAGTTCCTTTATCTGCAACAATCTCTTAGTCGACTCTAC
HiFi Gibson Assembly cloning of VAP27-1 and VAP27-3 DT1DT4 gRNA expression module into pHEE401E		VAP27-1 DT1 F	ATATATGGTCTCGATTGGGTCTACTTGCGAAGTTCTGTTTTAGAGCTAGAAATAGC
		VAP27-1 DT2 F	GTAGAGTCGACTAAGAGATTGTACTGGAGTTGTTCTCCCGGTT
		VAP27-1 DT2 R	AACCGGGAGAACAACTCCAGTACAATCTCTTAGTCGACTCTAC
		VAP27-3 DT3 F	GGTTAGAGACGAAGTAGTGATTGGATAATTATGTCGCCTTCAGTTTTAGAGCTAGAAATAGC
		VAP27-3 DT3 R	ATTATTGGTCTCGAAACTGAAGGCGACATAATTATCCAATCACTACTTCGTCTCTAACC
		VAP27-3 DT4 R	ATTATTGGTCTCGAAACAACGCAGTACTTCTTTGGACAATCACTACTTCGACTCTAGC
Restriction-ligation cloning of VAP27-4 and VAP27-6 DT1DT2 gRNA expression module into pKSE401		VAP27-4 DT1-BsF	ATATATGGTCTCGATTGCTAATGATGCAAGAGTCCCAGTT
		VAP27-4 DT1-F0	TGCTAATGATGCAAGAGTCCCAGTTTTAGAGCTAGAAATAGC
		VAP27-6 DT2-R0	AACCGCAAATACTGTGTTCGTCCAATCTCTTAGTCGACTCTAC
		VAP27-6 DT2-BsR	ATTATTGGTCTCGAAACCGCAAATACTGTGTTCGTCCAA
Genotyping of CRISPR target	TRB1 (DT1DT2)	TRB1 CrisprF	CTGCCATCATCTCCTCGATTTCG
		TRB1 CrisprR	CCAACCAAGTACACATCGCAAGATCC
	TRB2 (DT1DT2)	TRB2 CrisprF	GTTCAGTGATTATCTGCGACCATTCG
		TRB2 CrisprR	CTTAGCAAGATACATTCCGGAAACTATGG
	VAP27-1 (DT1DT2)	V27-1 CrisprF	GATGAGTAACATCGATCTGATTGG
		V27-1 CrisprR	CATCTCAGGAGTAACTTCCTTGG
	VAP27-3 (DT3DT4)	V27-3 CrisprF	GATGAGTAACGAGCTTCTCACCATCG
		V27-3 CrisprR	GAAGCAAGAACTTATCTTTACACTGC
	VAP27-4 (DT1)	V27-4 CrisprF	CGGCGTTGGCGAGAATCAG
		V27-4 CrisprR	CCATCACCATTTGTTGCTCCGG
	VAP27-6 (DT2)	V27-6 CrisprF	CATGACGACCGGAGATCTCGT
		V27-6 CrisprR	GGAAGCCATAGGAGAGTTGCCT
Sequencing of CRISPR targets		TRB1 seqP	CACATCGATGACCCTAGGCCTACC
		TRB2 seqP	GTGATGGTGATATATGCGCTGTAGC
		VAP27-1 seqP	GATCTCTTGCTCTCTCTATTTGACG
		VAP27-3 seqP	GATCCTGTCGACCTTCAATTCC
		VAP27-4 seqP	GAGAAGCAAAGCTACTGTGATC
		VAP27-6 seqP	GGTGGTGAGAAGTTGAGTTG
Restriction cloning of mTAGBFP2-HDEL into pB7WG2		HDEL-1	GGCAACCTCGCAAGGAGTGCGGATCCATGGTCTCTAAAGGTGAGGAGC
		HDEL-2	GAATGTACATTACAGCTCGTCATGGTTGAGTTTGTGACCAAGTTTTGACG
		HDEL-3	GACACTAGTATGAAGGTACAGGAGGGTTTGTTCGTGGTGGCTGTTTTC
		HDEL-4	GGTTTGTTCGTGGTGGCTGTTTTCTACCTTGCTTATACGCAGCTAGTC
		HDEL-5	CCTTGCTTATACGCAGCTAGTCAAGGGGCAACCTCGCAAGGAGTGC

Restriction cloning of pMDC83-mTAGBFP2	83 BFP-F	CTTGGCGCGCCATGGTCTCTAAAGGTGAGGAGCTAATCAAAGAG
	83 BFP-R	GGGTTCGAATCAGTTGAGTTTGTGACCAAGTTTTGACGG
Restriction cloning of Mito into pMDC83-mTAGBFP2	Mito-F	GGGACTAGTATGCTTTCACTACGTCAATC
	Mito-R	CAAGGCGCGCCTGGATCCGGGTTTTTGCTGAAG
Hot fusion cloning of FKBP-mCherry-TRB1$^{WT\ \Delta TMD}$ and FKBP-mCherry-TRB1$^{\Delta AIMs\ \Delta TMD}$	FKBP-F	GTCGACCTGCAGGCGGCCGCACTAGTACCATGGGAGTGCAGGTGGAAACC
	FKBP-R	ACCAGATCCACCACCAGATCCACCCTTGTACAGCTCCTCCATGCC
	TRB1 KSP-F	GGTGGATCTGGTGGTGGATCTGGTATGACGATAGAGCCGACGCAATCG
	TRB1 KSP-R	CATGGCCGCGGGATATCACCACTTTCAAAGAAGTATACCCGAAACTATAGC

Supplementary Table 2 The list of constructs used in this study

Name	Vector	Details
GFP-TRB1	pMDC43	N-terminal GFP and TRB1 full-length CDS fusion, 35S promoter
RFP-TRB1	pK7WGR2	N-terminal RFP and TRB1 full-length CDS fusion, 35S promoter
GFP-TRB1$^{\Delta AIMs}$	pMDC43	N-terminal GFP and TRB1$^{F21A, I24A, F279A, L282A}$ CDS fusion, 35S promoter
GFP-TRB2	pMDC43	N-terminal GFP and TRB2 full-length CDS fusion, 35S promoter
RFP-TRB3	pK7WGR2	N-terminal RFP and TRB3 full-length CDS fusion, 35S promoter
GFP-CsTRB1	pMDC43	N-terminal GFP and Citrus TRB1 full-length CDS fusion, 35S promoter
pTRB1: GFP-TRB1	Native promoter replaced the 35S in pMDC43-GFP-TRB1	
pTRB2: GFP-TRB2	Native promoter replaced the 35S in pMDC43-GFP-TRB2	
VAP27-1-RFP	Described as previous report, Wang P. et al. 2016	
VAP27-1-YFP	Described as previous report, Wang P. et al. 2016	
VAP27-3-YFP	Described as previous report, Wang P. et al. 2016	
VAP27-4-GFP	pMDC107	VAP27-4 and C-terminal GFP fusion, native promoter
VAP27-6-GFP	pMDC107	VAP27-6 and C-terminal GFP fusion, native promoter
CFP-ATG8e	pK7WGC2	N-terminal CFP and ATG8e full-length CDS fusion, 35S promoter
YFP-ATG8e	Described as previously reported (Wang et al., 2016)	
RFP-ATG8e	pK7WGR2	N-terminal RFP and ATG8e full-length CDS fusion, 35S promoter
Mito-mCherry	Described as previous report, Nelson B. K. et al. 2007	
Mito-CFP	Described as previous report, Nelson B. K. et al. 2007	
pMDC83-mTAGBFP2	Modified pMDC83	For expression of c-terminal mTAGBFP2 fusion proteins under 35s promoter. pMDC83 GFP CDS replaced with mTAGBFP2
Mito-mTAGBFP2	pMDC83-mTAGBFP2	mTAGBFP2 with N-terminal Mitochondrial localization signal described previously (Nelson et al., 2007), 35S promoter
GFP-HDEL	Described as previous report, Nelson B. K. et al. 2007	
mTAGBFP2-HDEL	pB7WG2	mTAGBFP2 with N terminal *At*WAK2 signal peptide and C terminal HDEL ER retention motif (both described by Nelson et al., 2007). 35S promoter.
pMDC43-BIFC-TRB1	pMDC43-nYFP	N-terminal sYFP-N and TRB1 full-length CDS fusion, 35S promoter
pMDC43-BIFC-VAP27-1	pMDC43-cYFP	N-terminal sYFP-C and VAP27-1 full-length CDS fusion, 35S promoter
ATG8e-AD	pGADT7	Y2H plasmid, ATG8e full-length CDS
TRB1^{WT}-BD	pGBKT7	Y2H plasmid, TRB1 CDS without transmembrane domain
TRB1$^{\Delta AIMs}$-BD	pGBKT7	Y2H plasmid, TRB1 $^{F21A, I24A, F279A, L282A}$ CDS without transmembrane domain
FKBP-mCherry-TRB1$^{WT\ \Delta TMD}$	pB7WG2	N-terminal FKBP-mCherry (Winkler et al., 2021), TRB1 CDS without transmembrane domain, 35S Promoter

FKBP-mCherry-TRB1$^{\Delta AIMs\ \Delta TMD}$	pB7WG2	N-terminal FKBP-mCherry (Winkler et al., 2021), TRB1 $^{F21A,\ I24A,\ F279A,\ L282A}$ CDS without transmembrane domain, 35S Promoter
EB1a-mTAGBFP2-FRB	Described previously (Winkler et al., 2021)	

Supplementary Table 3 The list of transgenic plants used in this study

Name	Details
Mito-mCherry	Mitochondria MT-RK marker line, Nelson B. K. et al. 2007
Mito-YFP	Mitochondria MT-YK marker line, Nelson B. K. et al. 2007
GFP-ATG8a	Autophagosomes marker line, Wang P. et al. 2019
GFP-TRB1	*35S*:GFP-TRB1 transformed in Col-0
GFP-TRB1 + MitoRed	GFP-TRB1 stable line crossed with MT-RK marker line
trb1/2 MitoRed	*trb1/2* (T-DNA double mutant) crossed with MT-RK marker line
vap27-1,3,4,6 MitoRed	Mito-mCherry marker transformed in *vap27-1,3,4,6* CRISPR line
pTRB1:GFP-TRB1	GFP-TRB1 expression driven by its native promoter in Col-0
pTRB2:GFP-TRB2	GFP-TRB2 expression driven by its native promoter in Col-0
pTRB1:GFP-TRB1/*trb1*	*pTRB1*:GFP-TRB1 expression in *trb1* (GABI_369F08)
GFP-TRB1$^{\Delta AIMs}$	*35S*:GFP-TRB1$^{\Delta AIMs}$ transformed in Col-0
GFP-TRB1/*atg5*	*atg5* crossed with GFP-TRB1 stable line
GFP-TRB1/*atg7*	*atg7* crossed with GFP-TRB1 stable line
GFP-ATG8a + RFP-TRB1	35S::RFP-TRB1 transformed in GFP-ATG8a marker line
GFP-TRB1 + VAP27-1-RFP	GFP-TRB1 stable line crossed with VAP27-RFP stable line

The full-length protein sequences of phylogenetic analysis in this study

***Adiantum capillus*_MBC9839808**

MDSSDGGDCKLDSHVGNPVALPLAREIYPSSSSEALEESSKLSSACEQAGSCHTPSADGSSCVFLASHDRPLEDLSRPC
PEQCLPDLGSSLTMASLSIEQAHCSSVEASSLHLADVAPTEQLWQEPCTQRAPSLSEGDCCLEEPLSSSSASQLSDGVIL
EEEDSPCNVQGPELSDDDKREMITEICKGVSVLSFRSEALGGSCYVYLVGTAHVSEESCREVQAAVQFLKPQVVFLELC
QKRTSVLQRTPFVVPTVSDMISTWKQRKTNLLGIVYSWFLAKVAAKLDVRPGAEFRAAYEEALACGAKVILGDRDIEV
TLRRTWGKMSAWYKTKFIFVLLYQSIFLPKADQLNELIEKLKDADILTIAFEELGKFSPTLMETVITERDLFMTAALQKIA
EHHSSIVAVVGRGHVAGITKYWGQDIPIHTLLEIPQKRSLLGLRLRTYMLIILVAFASVILYALH

***Adiantum capillus*_MBC9853572**

MGCLVTACLPSSNSAHLLMRQERDVSKRLLADRFPQLVSLVDDGTLLVLERSPCYQERRNNGFQEPMLLFLVGTSHFS
TVSALHVHNVVTAVRPQSVVVELCRSRAGLMNEIPDLNDATRPPSNLLSMSGKSFFAALGRSLGMGGQSAMTLRFLL
ALASEKLSSVAGVASGEEFRAAYKAAAEVDAQVVLGDRPIEITLRRAWEALDWVSRWKLTAFLLKGISSSKLDLSEERL
QVLRSDDALSFVFSYLSEEFPALLQPLIHERDRRR

***Adiantum capillus*_MBC9832533**

MWIRCGNLKYWTLHMQSLSCSDINVNKSMENRTSSTQPHAREVRRCSSVSCQYVSTSEQPVTYLMNSATKAKIYLV
GVNHISARSAEHVRHVIRHHRPNVVAVELCKERAVSLLLARKMKERSRRNFMSMKGSIKENLLKYSLEYFYSYIGNETD
GPLPGEELRVALEEGHAIGARFLFMDEDFKVTIRKMSKEFSLWEFLMFFLRNRKTHNQTYPNLHGAFLRIDQDNISKF
RDQTDCLLEPQVIEEAIAMGDAYFPGLTRALLHERNEHMVKGLRNMEGNVVAVVGAGHVPGMRRLWQEAEAQGE
EINASFS

***Amborella trichopoda*_evm_27.model.AmTr_v1.0_scaffold00037.16**

MTDGVSPNAEVLDTKLSHESHATTALSGKSMEIDVEEANKGEDIEPDMEEESDPPRFRGTLGLHESSNGGNDAELHD
GWQTYEKMELPEDLAKGVVLLNCESSVPGGTCDVYLVGTAHVSQESCREVQAIIRFLKPQVVFLELCPSRVSILTTQNL
KVPSMNDMIDMWRKKKMNALGIMYSWFLAKAAETLEVLPGSEFRIAYEEAVSYGAKVILGDRPVHITLRRTWTRMS
VWHRVKFLIYVLFQSIFMPSREELNKMLAQMNDADMLTLVIQEMSKVFPSLMETLVFERDLMKPEDGVYMEHHCE
GKRGSCYLEILGSS

***Amborella trichopoda*_evm_27.model.AmTr_v1.0_scaffold00069.102**

MERLASSLFLNPNPKPNFYQFHFPGFNLKISGKSYLSIMKSWVSIKPFFCASNSRVSITPPPLDFDYRAETFTDTIDTVSE
RYPELLDLVESGNLVLVKKMERGNGEPEMIWVIATSHVSVESVMGVSRLVKAVKPENVVVELCRSRAGIMYAPMESG
EVKASPKMNANVFSLSGSSFFAAVNRSLNLGGQSTLAFRLLLAAFSTKISSDIGRPLGNEFRAARKASEEVGAQIVLGD
RPIEITLGRAWGALNWREKLRLVGVLLRGINAPSLGMSENTIEASDSDGNISQIYKDLSVLYPSLLQPLIHERDVYLAWS
LKRSKAVKNTKSVVGVIGKSHMKGVIYALVSDQGSLRFRDLVGKRPSNPNGWVNSLLKSLARDTVLGFIGWALYERLK
SGLFAFGWTRTQVDQLPPLIRNVMLTEFEVWCKL

***Arabidopsis thaliana*_AT1G05270.1**

MTIEPTQSPSSEPEVHSGEDFVHIDDPRPTGDISLSDSIVNVEKDELLDEAAEEEFRGSDSVFSGGDGGGADDDGGEC
SSEATKVELPEELAKSVVILTCESTADGGSCDVYLVGTAHVSKQSCLEVEAIISILKPEVVFVELCSSRLSVLKPQTLKIPTM
SDMIESWKQKQNTFGILYGWFLAKIASHLEVFPGAEFRVAYEEAIKYGGKVILGDRPVQITLKRTWAKMPLWHKVKFL
YSILFQAVFLPGAEELEKMLKDMDNVDMVTLVIQEMSKEFPTLMDTIVHERDQYMASSLLRVASDHSSVVAVIGKGH
INGIKKNWKQPITMNDLMEIPSDKSVFTLKRIISSVAVAVAGTAIVSGILLSRRK

***Arabidopsis thaliana*_AT2G32340.1**

MELKPEVQYGDFSLTNSLVNVDKEDLRREEADSDAGSVIICDHSICDSGDGDICAVADYVECVAVETTEKLELPEEFAKS
VMVLTCESTAEGGSCDVYLVGTAHVSQESCREVEAVISALKPQAVFLELCSSRLSILTPQTVKVCCQEPPTWMEMIYM
WKKKHNLFEIVYGYILAKAAKKLEVFPGAEFRVGFEEANKYGGRVFLGDRSVQITLQRTWGKMPLWHKIKLVYSIVSQ
AVFLPNPKELEKMLKDLGDGDMLTLAIQEMSTEFPTLMETLVHERDKYMAYQLLRIASEHSSVVAVVGRGHLQGIKK
NWNQTINIKELMELPTNESIFTVKNILKYMVVVVVGTAIVSGMYLAK

***Arabidopsis thaliana*_AT5G52030.1**

MKLLPNPSLTLFPFFTPYTSKHIFRPIKVSVSVQTPPPDFDFRNDIASESRAAIAKTYPELLDLADNGTLILLQKQSFGPVP
AWRKEFVEPEAIWLVGTSHISPESASIVERVVRTVKPDNVAVELCRSRKVQFFIQTKFGAGIMYTSSVGGEVDQNLKS
GELSLTGTGFLGAVGRSLDLGGQTALALRLLLAVFSSKLSSVADRPFGDEFRAARKASEEVGAQLVLGDRPIEITLQRAW
NSLKWGEKFNLVMAVTRVITSSSGISAAELKEQETDENSGSLQLYERLSFSYPALLMPLIHERDTYLAWSLKRSKAVNG
CKTVVGVIGKGHMNGVIYALVSDSGDLRFRDLVGRGDSYNGGTGTTSNGWIQKVLKSFVRDTIIGFLLWELYEQYLM
MNQNLS

***Caenorhabditis elegans*_F38A5.2**

MSESSSNLNPDGTEFPDPNDVPPPSEDQLTEEDLFRMRSLTINSKKGLVGGASETAEQSIISLADSTSNFDEHVGDDAE
LDYELEQAPEATEDGAVVNNPVCGGYHFGRKRADDFVPNANTVTVLKWPTNLAIPPRNPVLSDSDEEWKKAILDSTI
YLIGTAHFSKESQEDVSNTIRAVQPDFVMLELCPSRISIISMDEARLLSEAKDLNSQKIIQTMKQNGAIQGILHVLLLSMS
AHVTRELSMAPGGEFRAAHRAAVATENCRVVLGDRPIQVTLQRALASLSIWQKIRFFFHVAFSHREKITAEEVERCKQ
RDLLEQLLAEMADDFPQLSQIFVEERDAYMTHALHMLVHRNSVEKRAQWLRGTTGQQFQPLTVVAVVGIGHTPGIV
NKWNTNIDFDPLMNIPPPSLGTKVFSTGVRIVVWGSLGYLAYRGGRAIFNRVSTFIESR

***Chlamydomonas reinhardtii*_CHLRE16g677800v5**

MVPSVATSSTTITQQPTHDPELVAELLKGPPEDFDFRAELTQGSREWVAANAPHLLDLVDDGTLLVVPRRHDYVERRS
DGYTEPRVVLLVGTAHVSRRSQLDVDRVIRAVRPDSVVVELCKSRSAALAAQPLALVEQQQQQQQQQHQQQNEA
ARSSGSGSDTDDGADRKASGLGTSTSSTSSSGTSGTSGSGTSTSTSTSGTSSSGNSASAPPGAYINPMNLGGGGGSAG
GGAGFAGAVARSVSLGGQSALLLRVLLAGLAKRTAGSLGVAGGGEFAAAQAAADAVGAQVVLGDRPVEITLSRAWA
ALGPLTRRAALCADLLKGAVGSPQQALSEELVERLKSDDAVSAFFKQLSSSYPELVAPLITERDLYLAWSLKRSKAVCGTG
TVVGVVGRGHLRGVAHALLRDRGGAGLRFSDLVEGRNSRRVRRRAAAEGAARLAAELALGAGAYVAWLAYNGEL

***Citrus clementina*_CICLE_v10015457mg**

MNRLSRLVTPINSPKSRRFFTFITTVKSQIPLRRGPTSLPLSLTKQFSPLLSTRITSFATANDPNFPKMDPNPPESGEEFVH
VDPLSESIVSLGDEVRDESSDRDETNGAVPVPEETKKVLPEELPRSVVILTCDSTAEGGTCDVYLVGTAHVSKESCREVE
AIIDFLKPQVVFLELCSSRVSVLTPQNLKVPTVGEMVDMWKKKHNTFGILYSWFLAKVASHLEIFPGSEFRVAFEEAM
KYGGKVILGDRPVQITLRRTWEKMPLWHKIKLLYSFLFQAFFLPSAEDLNRMLKEMDDVDMLTLVIQEMSKAFPTLLE
TLVHERDQYMSSTLLKVATEHSSVVAVVGKGHLQGIKNYWKQPVPVHDLMTIPSPKPAVSALKVLSSLGVAVAGVAIIS
GIYINCKK

***Citrus clementina*_CICLE_v10005112mg**

MEAFQKATFPIFSANPNLLSTKPIKPFKVSIKPPPQDFDFRTEILSDSQATIAKACPELLNLADDGTLVLIQKRQFGPVPA
WRSEFVEPENIWLIGTTHLSQDSAADVERVVRAIKPDNVVVELCRSRAGIMYTSNGGENDQQLRSNMFSLSGTGFF
GAVGRSIDLGGQTALALRLLLALFSSKISSDVNRPFGDEFRAARKVAEEVGAQIVLGDRPIEITLERAWNSLKWNEKLNL

LINVIRGITLPSDMSLDNLKEPSPDDSTFQLYKKLSFSCPSLLLPLIHERDTYLAWSLKRSKAVNNSKKVVGVIGKGHMN
GVIYALVSDQGNLRFRDLAGKRSSGDGSNGWIASLLKSLVRDTVIGILLWALYEQVKGTLS

***Citrus sinensis*_Cs2g21830P**
MNRLSRLVTPINSPKSRRFFTFITTVKSQIPLRRGPTSLPLSLTKQFSPLLSTRITSFATANDPNFPKMDPNPPESGEEFVH
VDPLSESIVSLGDEVRDESSDRDETNGAVPVPEETKKVLPEELPRSVVILTCDSTAEGGTCDVYLVGTAHVSKESCREVE
AIIDFLKPQVVFLELCSSRVSVLTPQNLKVPTVGEMVDMWKKKHNTFGILYSWFLAKVASHLEIFPGSEFRVAFEEAM
KYGGKVILGDRPVQITLRRTWEKMPLWHKIKLLYSFLFQAFFLPSAEDLNRMLKEMDDVDMLTLVIQEMSKAFPTLLE
TLVHERDQYMSSTLLKVATEHSSVVAVVGKGHLQGIKNYWKQPVPVHDLMTIPSPKPAVSALKVLSSLGVAVAGVAIIS
GIYINCKK

***Citrus sinensis*_Cs9g16830P**
MEAFQKATFPIFSANPNLLSTKPIKPFKVSIKPPPQDFDFRTEILSDSQATIAKACPELLNLADDGTLVLIQKRQFGPVPA
WRSEFVEPENIWLIGTTHLSQDSAADVERVVRAIKPDNVVVELCRSRAGIMYTSNGGENDQQLRSNMFSLSGTGFF
GAVGRSIDLGGQTALALRLLLALFSSKISSDVNRPFGDEFRAARKVAEEVGAQIVLGDRPIEITLERAWNSLKWNEKLNL
LINVIRGITSPSDMSLDNLKNSVSRMLAAISSTIL

***Cucumis sativus*_Cucsa.072860.1**
MVAYAIIMEGRKVVFEEGKFESWFISFLHGKKGKARCMNRLTRCATQLNLTESHRFIVTATVKPHRRRLNYALTFKPHR
HFSAPFDFGIFFSQNSDRPRRLPRLPQVMDPVPPESDSPAVEDFVHIEDPNIESLCESIVSTTDEQINDEAASVISPEAEE
GLAEQRRVLPEELSRSVLVLTCETTGEGGICDVYLVGTAHVSQESCREVQAVISYLKPQVVFLELCASRVAVLTPQNLKV
PTMGEMVEMWKKKHNIFGILYSWFLAKVANKLEVFPGSEFRVAYEEAMKYRGKVILGDRPVQITIRRAWAKMPLW
HKIKLLYSFVFQAFFLPSPEELTKMLKDMDDVDMLTLIIQEMSKEFPTLMDTLVHERDRYMSTTLLGVAKEHQSVVAV
VGKGHLSGIKKNWQQPVTVGFTLPFLEKFDWYSLSSLYTSIHFLKIAARGSFGNASPKRSPCHQDLFVNRCCSCWSCH
HIRCLPCMQKITLLKLTSKANLMQRMLA

***Cucumis sativus*_Cucsa.251010.1**
MEALKSTAIPIFKSSPLSFPTKSLKPINVSINPPPSDFDFRLEISRDSRATIAETHPELLDLADDGILVLVDKTKFGPVPAWR
AEFVEPQAIWLVGTSHISPKSVKDVERVVRAVKPDNVVVELCRSRAGIMYTLDTGEPDQKLKSNMFSLSGDGFLGAV
VRSINLGGQTALALRLLLAVFSSKISSDVNRPFGDEFRAARKASEEVGAQIVLGDRPIEITLERAWNALTWTEKLSLVSSV
IRGITSESDFSQNIDEESDGNGSSLQLYEKLGFSYPSLLQPLIHERDTYLAWSLKRSKAVNKSKRVVGVIGRGHMNGVIY
AITSDQGNLRFRDLAGKKAGEGNSNGFVSSLLKDLVRDTIIGVVVWVLYEQLKQLHIIP

***Cyanidioschyzon merolae*_CMT494C**
MSTVAVRARWLSGIWRWRGFRQFVRSEQWWRREPRPAAYRTLVPQAQPAFRVWVSGSLSTSAGNSTFGSAPGDE
TTNSTGTAAALTDDNPLRLVNGSTGAVAHVLGTAHISALSVSQTRELIRRVQPDTVVVELDDERVQTVRERLDKKDPS
QDSSLFSELLRVFTDPRGGSLGSRMFEVYFKLMYRALRMAGFLPGAEFVAAIEEAERIGARVVLGDRNIHETMERLRA
AVFHNFSDVLHALVSPPPPELESLLDNLKDQARGGNARKISTDFVDALLDRKSVRLLTRFLSRSLPAVSKVMLDERDVIL
ASKIATAPGQKIVAIVGAAHLDGIERWWLQNIEPDPIIVSSSPLPSQS

***Danio rerio*_NP997788**
MEQDNNSEDDSVGPAEDDTQPPFPLGLSDSEAVEVLWQVRTQRRQKEPDLPETVTRLTTPEGSVVYLVGTAHFSDSS
KKDVATTIRAVQPDVVVVELCQYRVSMLKMDEKTLLKEAKDINLEKVQQAIKQNGVMSGLMQILLLKVSAHITEQLG
MAPGGEFREAFKEAGKVPFCKFHLGDRPIPVTFKRAIAALSLWQKARLAWGLCFLSDPISKEDVEKCKQKDLLEQTMS

EMIGEFPALHRTIVAERDIYLTHTLRQAARCVEAPPTAEKVPAVVVGVVGMGHVSGIERNWDKELNIHEIMSVAPPSR
LGRVVRTVLKGAMWGLLGYACYRASKGVGRALLSLPTVQSLLQNIRTPSV

***Desulfobacterium vacuolatum*_SMC84642**
MDANAHDKENMIDRLSFNGKKITLIGTAHVSRESARIVKEIIQKEKPDTVCVELCNSRLKSIKDNDSWRNMDIVNVIK
EKKAMLLLMNLMLASFQKKIAEKFDIKPGQEMINAVAAAEEQGATLIPADREIQVTLTRVWRNMGFWEKIKLMFQL
MLSMGSSDEISEEEIEQMKQEDLLQTLLADVKKSHPIMEKVLIAERDQFLAAKISNAPGNHIVAVVGAGHAPGIKKHL
APETPIDTDTLTEIPPAGKSGKILKWMIPGLIIVLMALGFFMKGAEAGTDMIWLWIAANGIFAGIGAIVALAHPYTIISAI
AAAPLTSLNPMIAAGWVSGLVEAVSRKPKVRDLESIPKDILSIKGFWRNNVTRILLVVVFTNLGSTIGTMTAIPLMLKVI
N

***Drosophila melanogaster*_CG46280**
MDVSASTSFESSPDKRNSIYVGDNTFYDSALDHQLSTTAYKSCNESLISELSQNPLGPSNSANKTASAEFGNESAAFKPL
GSPAAFNNATPALNASMLLIQSESTDTNTSQEEVDPKSQLANKTIFKTDNPNLSIIEINDNSIKEEDLEKVVLVEGDSVK
NLLKKSPSKEASANADKRRRKESLLHTSKQKLDISIAEASAAADGDGEKRDLVSVIDQPQTKREITIYDTIEEFEQNLPST
VTLLNTPFGSKVYLVGTAHFSEESQDDVSYVIRNVRPDVVMVELCPSRIHILKLDEKTLLEEAKSINIPKIRGILHTHGYIN
GIFFILLLQMSAQIAKDLGMAPGGEFRRAFEEIHKLPGCILHLGDRPIRITLYRALRALSMWQTMKLVWRLTFTDSISIE
EVEECKQSDLLEKLMQEMAGEFPAFSDVFVRERDVFLCHSLQLAALPQAAPGGQQVRPVRVVGVVGIGHANGIAK
MWGTVDPKKIPAILEIPPASLGQRVCKYTLKYGLIGLGCYGAFRFFRPRLTRLF

***Fragaria vesca*_mrna23910.1-v1.0-hybrid**
MDLEPAVPDPNSGEDFVHVENSLSDSLTESIVNVERELEENDDVSNSVVAEEDFDNGRTTVLPEELSRSVMVLTCAST
AESGVCDVHVVGTAHVSAESCREVEEVIRYLKPEVVFLELCSSRVAVLSPQNLKVPTAGEMIDMWKKNHNAFGILYG
WFLAKVASKLEVFPGAEFRVGYDEAMKYGGKVVLGDRPVQITLRRTWAKMPLWHKIKLIYSILFQAVFLPDPEELNK
MLKDMDDVDMLTLVIQEMSKEFPTLMETLVHERDQYMSSTLLRIAQEHNSVVAVVGKGHLQGIKKHWEQPVVVTD
LMGIPSQKTGFSMVKVLKSCGVVVAGAAIISGIYLASKK

***Fragaria vesca*_mrna13326.1-v1.0-hybrid**
METIRSTFPIFHTNPVFFTRKPLRPIKVSIKPPPPDFDFRSEILEDSRATIAKTHPELLDLAENGSLFLVDKSRFGPVPAWR
TEFVEPEAIWLVGTTHISQVSASEVERVVRAVKPDNVVVELCRSRQPIFRAGIMYASMDDESGQQLKSNMFSLSGNG
FFGAVGRSINLGGQTALALRVLLAVFSSKISSNINRPFGDEFRAARKASEEVGAQIVLGDRPIEITLERAWNSLTWTEKLS
LVTSVFRGITSSSDMSQMSLKESSTSDSTFQLYEQLSSSYPSLLQPLIHERDTYLAWSLKRSKAVNKSKRVVGVIGKGHM
NGVIYALLADSGDLRFRDLVGGQRPSWSSNDAASNGWVVKLLKDLVRDTIIGIVLWAFYEQIKGGFIE

***Gallus gallus*_NP001025903**
MQEEQQPEAAADPMSSSDVPEDGPKETSSVSQSISDADAFKILLEMKMKKRQKKPALPSTVTELNTEDGSKVYVVG
TAHFSDSSKKDVVKTIQEVQPDVVVVELCQYRVSMLKMDEKTLLKEAKEINLEKLQQAIKQNGVMSGLMQMLLLKV
SAHITEQLGMAPGGEFREAFKEASKVPFCKFHLGDRPIPVTFKRAIAALSFWQKVKLAWGLCFLSDPISKDDVEKCKQ
KDLLEQMMAEMIGEFPDLHRTIVSERDIYLTYMLKQAAKQIELPRASETEPQRYIPAVVVGVVGMGHVPGIEKNWNS
DLNIQEIMSVPPPSASTAQSYLQKLTEIRSQH

***Halalkalicoccus paucihalophilus*_WP066382380**
MTDRQVPRGAEGGSVRLVGTAHISKDSVTEVEETIERERPDVVAVELDEGRYRQMKGELPDDLDAGDLLRGNTVFQF
LAYWMLSYVQTRLGERFDIEPGADMHAAVETAESLGLGVALVDREIQTTIQRFWARMTLVEKLKLVGGLFLGLFGYGT

DEEELDVEELTDADVVTAMMAEFRRFSPGGAEALIDERDAYIAHKLLALRTAGHDVIAVVGAGHREGIERYLADPESLP
PMDSLVGTQKGRRFSIYKAVGYLIALGFLVFFFLLVMAGAQNDFLLRVFATWFLFNGVFAFALAKLAGAHWPSATVGG
AIAWMTSVNPLLAPGWFAGYVELRYQRVNVADIGTLNEILADEESPIRDLFGRMLDVPLFRLIAVVAATNIGSIIATLLF
PLVVLPWLANDIGGVGAIGDLMIEGARNSAELIWGVFA

***Homo sapiens*_ENSG00000269113**
MDGEEQQPPHEANVEPVVPSEASEPVPRVLSGDPQNLSDVDAFNLLLEMKLKRRRQRPNLPRTVTQLVAEDGSRVY
VVGTAHFSDDSKRDVVKTIREVQPDVVVVELCQYRVSMLKMDESTLLREAQELSLEKLQQAVRQNGLMSGLMQML
LLKVSAHITEQLGMAPGGEFREAFKEASKVPFCKFHLGDRPIPVTFKRAIAALSFWQKVRLAWGLCFLSDPISKDDVER
CKQKDLLEQMMAEMIGEFPDLHRTIVSERDVYLTYMLRQAARRLELPRASDAEPRKCVPSVVVGVVGMGHVPGIEK
NWSTDLNIQEIMTVPPPSVSGRVSRLAVKAAFFGLLGYSLYWMGRRTASLVLSLPAAQYCLQRVTEARHK

***Methanosarcina barkeri*_WP011306000**
MSKPEITDSQDKDPDYNFHSSSQESIYSMDKLVTESAENFTSVGKSKIPGSSINMPQEEKTSVPASKIEPDELETKLETK
LETKLDISSEIIAESIPDSASEVSIPSSSQFSDAHQAEYQPSKIVLIGTAHVSEKSVAEVKAAIRDLKPDIVAVELCRGRYDSL
KGNVQEKQVPIKDILSEGKVNYYVIHWLLAYVQKKIGDDMGVRPGAEMLSAIEEAESIGAKVALIDRDIQVTLQRFWG
KMKFLEKVKMIGSLLGGLIGIGKGTEIDIDKITETDVVTGLVNELRDFAPTAAEVLIDERDAYLAGSILNVAAGGNKTVV
VVIGAGHKPGVTKYLKNPKSVPSLSNLMQVPKKRIGLGKIVGFAFVAVIVGFFLILLLSGVPLKLLLIAFGWWFIITGTLS
AIGTLLAGGHPYSVLTAFSVAWLTTLHPLIAAGWFAGLVEAKQRNPTTADIKALAGVETFREMFKNKFMRVLLVASFA
NIGSMTGTFVAAYVMLHVTGIDPRDVLLSGFNALGL

***Methanosarcina barkeri*_WP048109790**
MIKPEITDSQDRDPDYNFHISSQESIYSMDKLVTESEETLTSVGKSKISGSSVNMPQEEKNSVPASKIEPNELETKLDISS
ELITEPIPDSASEVSVPSPSQFSDAHQAECQPSKVVLIGTAHVSEKSVAEVKAAIRDLKPDIVAVELCRGRYDSLKGNVQ
EKQVPIKDILTEGKVYYYIIHWLLAYVQKKIGDDMGVKPGAEMLAAIEEAESIGANIALIDRDIQVTLQRFWGKMKFPE
KIKMIGSLLGGLIGIGKGTEIDIDEITKTDVVTGLVNELRDFAPTAAEVLIDERDAYLAGSILKVAAGGNKTIVVVIGAGHK
PGVTKYLKNPKSVPSLHSLMQVPKKRISLGKIVGFAFVAVIIGFFLLLLLSGVPLKLLLIAFGWWFIITGTLSAAGTLLAGG
HPYSVLTAFSVAWLTTLHPLIAAGWFAGLVEAKQRNPTTADLKALGEIETFKEMFNNRFMRVLLVASFANIGSMTGTF
VAAYVMMHVTGIDPRDVLLSGFNAIGL

***Methanosarcina barkeri*_WP048122625**
MSKPEITDSQDKDPDYNFHSTSQESMYSMDKLVTESAENSTSVGKAKISGSSINMPQEEKTSVTASKIEPDELETKLDI
SSEIIAESIPDSASEVSIPSSFQFSDAHQAEYQPSKIVLIGTAHVSEKSVAEVKAAIRDLKPDIVAVELCRGRYDSLKGNVQ
EKQVPIKDILSEGKVNYYVIHWLLAYVQKKIGEDMGVRPGAEMLSAMEEAESIGAKVALIDRDIQVTLQRFWGKMKF
LEKVKMIGSLLGGLIGIGKGTEIDIDKITETDVVTGLVNELRDFAPTAAEVLIDERDAYLAGSILRVAAGGNKTVVVVIGA
GHKPGVTKYLKNPKSIPPLDSLMQIPKKRIGLGKIVGFAFVAVIVGFFLLLLLSGVPLKLLLIAFGWWFIITGTLSAIGTLLA
GGHPYSVLTAFSVAWLTTLHPLIAAGWFAGLVEAKQRNPTTADLKALAGVETFREMFKNKFMRVLLVASFANIGSMT
GTVVAAYVMLHVTGIDPRDVLLSGFNALGL

***Oryza sativa*_LOC_Os05g42010**
MIRAPRRLLPDSSSPPIHRRRLPLPPLFRRRLSPLQASLVQALLCTGPIRGGRGLTPLGLLMDPAADGEGGGYEDASEFA
DAETGGGEVVRGEGEGERERKELPEELAKGVVCLECETSPEAEAAGAGGTCRVYVVGTAHVSQESCDQVKAVIDYLK
PQAVFLELCASRVAILTPQNLQVPTMNEMIDMWKKKKMNTFGILYSWFLAKVASQLDVLPGAEFRVAFEEAMSYGG

KVILGDRPVQITLRRTWGRMSLWHRAKFLYYIVFQSIFLPSPEELNKMLKDMEDVDMLTLVIQEMSKAFPTLMETLLH
ERDMYMSSKLLKVAKEHSSVVAVVGKGHVSGIKKNWEQPIEIESLLVLPVTKQGASKMKILASIGALGGVVIATGIYIW
SRK

***Oryza sativa*_LOC_Os08g43230**
MYVTTDSSAGEPLLKSNMFSLGGSKFFGAVNRSINLGGQTALALRLLLAVFSSKISSGANRPFGEEFRAARKVSEDLGA
QLVLGDRPIEITLERAWKSLSWDEKTKLVVSLFRGITSTTDTSQDEKAAGSPYELYEKLSISYPSLLQPLIHERDMFLAWS
LKRSKAVNKSKTVVGIIGKGHMNGVVYALISDQGDLRFRDLVGRASSDTWASSLIKGLVRDTIIGIVLWGLYEQLHAVF

***Physcomitrella patens*_Pp3c16_8270V3.1**
MSFESGDIDELGNHVVEDTSSDLNLPNISVDLSENEFPSKLANATEHLEIGEPEEHRTEFSPDTASADLSRTDSLTDDTD
AASSSTLASEAPERLETPRTEEHVKKFVAEVTKTIVSLCNEDSNARSPSDTVTIPERLQSDEVVEAKGLTYAEIVSADPVL
PDSSVYTLDAERLNSEGCDEAEGRTYAEVASAERAVPDSMTNINDGERLDADEGEKKKCRTYAEVSKENPILRDSFTD
SAGAVSLSDVAASTESDETRCSEEASREGVAVEVLELGAGVGDGTQKAMREGGELEEHIHDKGLEGVREEVSKEGDH
VEELVLGSGVKDASKGSSSPHEEGIQSATDTVQGTSTNASENNKPRPKSVFHNIAENEAREEPHLDKTAISSTLQGEAE
YLPNLKQLPAETGKNVLQLKAESSANDGVCLVYVIGTAHVSKASCEEVQALIRHVKPDVVFLELCSSRTNILLPRKNKVP
TVSGMLESYKKKEMNVFGLLYSWFLAKVGEKLEVLPGTEFRVAYEEAVRCGASVTLGDRPVQITLKRTWGSMSLWLK
TKFLFSVLTATFNMPSAEEFHALLEKMDESDELTLMVQELSKTFPTLLQTLVNERDLYMVANLRNVAQCRSSVVAVVG
RGHLSGMSKHWEEDINVEELLTLPSKKPARRLWLWSTVALGVGGVAVGIHVLRRR

***Physcomitrella patens*_Pp3c25_14680V3.1406**
METVSQPWRTSLSFSASIPYTSVMGFRIQKYYAYSWKKPVRLPQICCHSSTRVDGSQKVRPPPSDYDFRAETSDGTQE
MVQRLYPELMDLVEDGTLVIVKRPPDYVERRSDGYVEPEVVYIVGTAHMSKLSANQVTRVINAVQPENVVIELCRSRA
GIMYDEAQTSDVKQGSKNLMSMSGDNFGSAVGRSLKLGGRSALALRLLLAGVSKRLSASAGVATGEEFRAARKAAEA
LGAQIVLGDRPIEITLQRAWRSLKWDERLRFGATLVQGMSDKNLNVSEESLQMLKSDDALSAMFGELSSRFPSLMQP
LIHERDMYLAWSLKRSKAVNGCKRVVGVVGKGHLRGIVHALIHDQENLRFKDLVGSRGIGTENSKGEKLRKFATNLAI
ETVIGLLTWWAWESFQHH

***Physcomitrella patens*_Pp3c3_37060V3.1496**
MIEGQMRNRMMDQRSLQDRDMMLLRELERRLRAAAAQGDTTHQTDIDELIKNLEGRLRPMKENIENETTEREKTS
PEQPGYSTTYDYLNSWLKGWDHCLKEEELRLRKQRKETKNEQQPGVSSDRDVKSEGSASTSKEPCEAKTEDSSTTLKE
KDHVKNSSEQPDVIRGDSSTTLKEDDQVYTPQDLDVKAENSSSDLKEQDQVYTPEMIQEIEDKKKRLLEEEFKKQNVE
RKKAEAPNSKQAPSDGTVVVYLRNEENGADLYLVGTSHVSQQSADEVRDVIRRVKPDYVLVELDRKRYNSMLQRQNG
QNNPFAFVQQMVETLTNNNIGAVGKVLGLGLSGFYWLLAYWGLQPGQEFKVAIQEGKKAGAKIVLGDQDIDVTLKH
FGEQTSVAEALQFFAQPMPSDIAQKMSGTTPRAPRQMEFEQLRDRKLVRQLNEEMEKKAPALTNILLRERNESMTK
ALRNLSGKVVAVVGLAHLDGIEQLWREANEKYLNSTE

***Physcomitrella patens*_Pp3c12_24810V3.1291**
MLVAARRAARTLLVDSSTFQSSSCRHRHLVAAVLVARESFCSASSTITEDGSVSYLRNPRNGAQVYLVGTAHVSVKSAD
QVREVIQRVRPDRVAVELCQERAKNLMSDNPQRKGKTPLQQLQELFNLPGGLGQKLIGFWMKSMYELIRNTGVEP
GKEFRIAMEEAQRLNAEILYIDQNVHETIKRLRDVITIWDVLKMLKNPNQHLDTYPSFMKDMEHRDFEETVERVKTR
ENVREMMTWMEQSFPALVKVMVHERDQLMVKRLLECEGTVVGVVGMAHMDGIERLWKEAE

***Picea sitchensis*_ABR18005**

MDDNNAKNDDYSDNHKSQPEEEVEQPAVIGGGGEESGFEDFVDIDRSEAVADIGLEDQPSVTLHSLLAETPVDHND
LITEGSGIEPKPEATFEKDTETVGSSETPDNGEVPADNGAPDECEGGFSAESSARDQPEDDFHETGPDSEERGLPEEYS
KGLVVLQCESRTPSGICNVYLVGTAHVSLESCREVQAVIHFLKPQVVFLELCPSRVAMLVPQNLEVPSIREMIDMWKN
RHINAFGVLYSWFLAKVAAKLEVFPGSEFRIAYEEAMSYGAKVILGDRPVQITLRRTWAKMSLWHKTKFLFCMFFQAI
WLPSPEELNKMMGDVDVLTLVIQEMSKTFPSLIETLVNERDLYMSSTLLKVAKEHDSVVAVVGRGHLSGIEKNWMKP
VSVNSLLEVPVAKSSSMKLWTAVALAVGSVAIFTGLYLTRKK

***Populus trichocarpa*_Potri.007G121900.1**
MNRLTRSQLSLLNSPDSHRFFTTITTKLPLRHPQTTFNFSHSIYSRRKFPQIITFATKKLTNPIIADPNRNPMDPNPSKED
FVHIESPSNNDNHLSESIVDVANELSEDDNKNDVVMGRKELPEELSRSVMVLTCESKAEGGTCVVHLVGTAHVSQES
CREVQAVVSYLKPQVVFLELCASRVAVLTPQNLKVPTMGEMIEMWKKNHNTFGILYSWFLAKVSDKLEVFPGSEFRV
AFEEARKYEGKVVLGDRPVQITLQRTWGKMPLWHKVKLLYSLLFQALFLPSSEDLDKMLKEMDDVDMLTLVIQEMS
KQFPTLMDTLVHERDQYMSSTLLRIAKEHTSVVAVVGKGHLQGIKKHWGQPFEMKDLMEIPSQKPAVSARKVLASL
GVAVAGVAIVSGFYLSRKK

***Populus trichocarpa*_Potri.007G121800.1**
MKTRYVKRMTRSLLTQLSSPHSHRFFSSTTTTKSPLRHHQTTLISTRPTYSPPKFLRITTFATKNESIFDAADELREDNNK
HDVASERKEPLHEFSRNVVVLTCESKAEGGQCVVYLVGTAHVSQASCREVEAVIRHVKPQVVFLELCASRVGLLTIRNL
KVPTMKEMIEKWKKTQNAPQIFLSWFYATVGDKLGVVPGSEFQVAFEEARKCEAKVVLGDRPAQITFRRTQGKLPF
WHKVKFLCAVFVQTLFSSSSKSIDTMIKDLDDVKKVTISTKKLSKQYPTVMETVVDERDQYMSSILLRIAKEHTSVVAVI
GKGHLQGIKKYWEQPIELKDLLELPPQKPPFSALKVLAVAGMAIGSAFIFQARNS

***Populus trichocarpa*_Potri.017G038500.1**
MDPNPSNEEFVHIENPSDNDNHLSESIVDVANELSEDDNKNDDVVERKELPEELSRSVMVLTCESKAEGGTCVVHLV
GTAHVCQESCREVQAVISYLKPQVVFLELCASRVAMLSPQNLKVPTMGEMIKMWKKNHNTFGILYSWFLAKVADKL
EVFPGSEFRVAFEEARKYEGKVVLGDRPVQITLRRTWGKMPVWHKVKFLYSLLFQALFLPSSEDLEKMLKEMDDVD
MLTLVIQEMSKQFPTLMETLVQERDQYMSSTLLRIAKEHNSVVAVVGKGHLQGIKRHWEQHIELKDLMELPSQKSAV
SAWKVLASLGVAVAGVAIVSGIYLSRKK

***Populus trichocarpa*_POPTR_012G133900v3**
METLLKIPFPIFSNNPHILTTKPTKPLKVSIKPPPPDFDFRSEILQESRATISRTYPELLDLANDGTLLLIEKKLFGPVPSWRT
EFVEPEAIWLVGTTHISSQSAAEVERVVRAVKPDNVVVELCRSRAGIMYISDEGEVGQQLRSSMFSLSGTGFFGAVGR
SINLGGQTALALRLLLALFSSKISSDVNRPFGDEFRAARKVAEEIGAQIVLGDRPIEITLERAWNSLKWREKLSLVIAVVR
GITSSSDISKNNFKASSTDDRTFQLYEQLSFSYPSLLQPLIHERDTYLAWSLKRSKAVNNGKRVVGVIGKGHMNGVIYAL
ISDQGNLRFRDLAGRRSSGDDGSNGFVAGLVKSLVIDTAIGILLWQLYEQLKGSL

***Solanum lycopersicum*_Solyc07g052560.1.1**
MVDMWKKNQNPFGILYSCYQLEVVPGGEFRVAYEEAMKYGGKVILGDCPVQITLGRTWAKMQLWHKTKFLSSLLF
QALFLPNTEDLNKMLKEMDDVDMLTLAIQEMSKRFPTLWTTLVHERDLFMSSTLLRVAGEHNSVVAVVGKGHLPGI
KKSWKQPIELNELLTIPSQKRAISVIKSCQHLELQSAIISGIYTSIKK

***Solanum lycopersicum*_Solyc12g005780.1.1**
MLRRLTRQLTFLNSAESNRFNSLTGSPYRRHFPVKSLPVNFPVVGPSSLSISVKCKSSSSYSAVIGRRMPVEMSSSPMTA
SVVEDFVHVDGEVANLNSEGSEASVVEEQGAEEVISANVEGEGEGYERKVLPEELSRSVMMLTCDSSANGGICDVYV

VGTAHVSSESCQEVEAVINFLKPEVVFLELCSGRVGILTPQNLKVPTMGEMVEMWKKKQNLFGILYSWFLAKVATKLE
VFPGAEFRVAYEEAMKYGGKVILGDRPVQVTLRRTWAKMPLWHKTKLVYSLLFQAVFLPKPEDLVKMLKDMDDVD
MLTLVIQEMSKQFPTLMDTLVHERDQFMSSMLLKVAREHSSVVAVVGKGHLPGIKKNWEQPIEVKELLSIPSPKPLIT
VSKIVTTLGVAVAGVAIISGIYVSSKK

***Solanum lycopersicum*_Solyc03g026260.2.1**
MKSCNVATPLFTKRQPLEPHYHSILLLLLLLLAPAMRILTSPFPISPPIIPRTKFHQCRVAINPPPPNFDFKSEFFSASRDAI
EDTHPELLDLADNGTLFLIKKNQFGPVPSWRTEFVEPEAIWLIGTNHLSMESPLHVERVIRTVKPENVVVELCRKFRA
GIMYTSDDSDLNQPLKSNMFSLSGNGFFDAVGRSINLGGQTALALRIVLALFSSKMSSSANRSFGDEFRAARKAAEDI
GAQIVLGDRPIEITLERAWTSLKWKEKTSLLLSVFGGITSSTELSTKALKESSSDDSNFQLYEKLSFSYPSLLQPLLHERDTF
LAWSLKRSKAVNNCKQVVGIIGKGHMNGVIYSLVSDQGNLRFRDLAGKSPSGGLSGWTTTIFGNLVRDTIIGVLLWLL
YEQLTSGLKLID

***Zea mays*_GRMZM2G089962_T01**
MIRAPRLLAAHAPSPIRHRLSLAPISTIYRRLPWSPRAAFLAAQAPISTTRLASSSRLVPLLALPMDPAAPEPVPAPATDT
DAETNGYEDASEFEDAEAGGEGAGAGTETGTGTGSGSGTMPAGGVAEMKELPEELAKGVVCLECETSAEAAVSGVG
GTCRVYVVGTAHVSQESCDQVKAIINYLKPQAVFLELCMSRVAILTPQNLQVPTMNEMIDMWKKNKMNTFGIIYSW
FLAKVASQLEVLPGAEFRVAFEEAMSYGGKVILGDRPVQITLRRTWGKMSLWHRAKFLYYIIFQSVFLPSPEELNKMLK
DMDDVDMLTLVIQEMSKAFPSLMDTLLHERDMYMSSTLLKSAREYSSVVAVVGKGHVSGIKKNWQQPIQVNCLLEL
PDANQGPSKLKILASIGALTGVIIASGIYVWGRK

***Zea mays*_GRMZM2G159581_T01**
MRSRPASMAAGPTPSAAAASLRPPPPCFDYRAAVLADTRAAAAGNPALAGLIESGALVRVRRRFGPVPAWRPPDFV
EPEDVWILGTSHLSRDSVADVERVLRAVQPDNVVVELCRSRAGIMYASSDSSDEPLLKSNMFSLGGSKFFGAVSRSIN
LGGQSALALRLLLAVFSSKIASSANRPFGEEFRAARRVSEDIGAQLVLGDRPIEITLERAWKSLTWDQKAKLLISLFRGITS
KTHTPQDEKAAVSPYELYEKLSTSYPALLQPLIHERDMFLAWSLKRSKAVNKSRTVVGVVGKGHINGIVYALISDQGDL
RFRDLVGRASSDTWVSSLVKGLVRDTVIGLVLWALYEQLQAVL
