# Supplementary material for: TraB family proteins are components of ER-mitochondrial contact sites and regulate ER-mitochondrial interactions and mitophagy
Source: Nat Commun. 2022 Sep 26;13:5658. doi: 10.1038/s41467-022-33402-w (PMC9513094; doi:10.1038/s41467-022-33402-w)
Supplement: Supplementary file 2 — Reporting Summary [file 41467_2022_33402_MOESM2_ESM.pdf]

nature portfolio

Corresponding author(s): NCOMMS-21-41874A, Pengwei Wang

Last updated by author(s): Apr 10, 2022

Reporting Summary

Nature Portfolio wishes to improve the reproducibility of the work that we publish. This form provides structure for consistency and transparency in reporting. For further information on Nature Portfolio policies, see our Editorial Policies and the Editorial Policy Checklist.

Statistics

For all statistical analyses, confirm that the following items are present in the figure legend, table legend, main text, or Methods section.

n/a	Confirmed	
☐	☒	The exact sample size (*n*) for each experimental group/condition, given as a discrete number and unit of measurement
☐	☒	A statement on whether measurements were taken from distinct samples or whether the same sample was measured repeatedly
☐	☒	The statistical test(s) used AND whether they are one- or two-sided<br>*Only common tests should be described solely by name; describe more complex techniques in the Methods section.*
☒	☐	A description of all covariates tested
☒	☐	A description of any assumptions or corrections, such as tests of normality and adjustment for multiple comparisons
☐	☒	A full description of the statistical parameters including central tendency (e.g. means) or other basic estimates (e.g. regression coefficient) AND variation (e.g. standard deviation) or associated estimates of uncertainty (e.g. confidence intervals)
☒	☐	For null hypothesis testing, the test statistic (e.g. *F*, *t*, *r*) with confidence intervals, effect sizes, degrees of freedom and *P* value noted<br>*Give P values as exact values whenever suitable.*
☒	☐	For Bayesian analysis, information on the choice of priors and Markov chain Monte Carlo settings
☒	☐	For hierarchical and complex designs, identification of the appropriate level for tests and full reporting of outcomes
☒	☐	Estimates of effect sizes (e.g. Cohen's *d*, Pearson's *r*), indicating how they were calculated

Our web collection on statistics for biologists contains articles on many of the points above.

Software and code

Policy information about availability of computer code

Data collection	The collection of plant phenotype and microscopic statistical data were performed using Image J (v1.53); Microscopy image were captured using Leica Application suite X; qPCR data was obtained using Roche Light Cycler 480; Proteomics was identified by LC-MS/MS using an LTQ Orbitrap Velos (ThermoFisher Scientific); All software used in this study are either commercially available or open source.
Data analysis	All statistical graphs were performed using the GraphPad Prism software (v7.00); Evolutionary analyses of Phylogenetic tree were conducted in MEGA771 (v1.0.5877); The Mascot search engine (version 2.4.1, MatrixScience, http://www.matrixscience.com/) was used for MS-based protein homology identification against the TAIR10 database; All software used in this study are either commercially available or open source.

For manuscripts utilizing custom algorithms or software that are central to the research but not yet described in published literature, software must be made available to editors and reviewers. We strongly encourage code deposition in a community repository (e.g. GitHub). See the Nature Portfolio guidelines for submitting code & software for further information.

Data

Policy information about availability of data

All manuscripts must include a data availability statement. This statement should provide the following information, where applicable:

- Accession codes, unique identifiers, or web links for publicly available datasets
- A description of any restrictions on data availability
- For clinical datasets or third party data, please ensure that the statement adheres to our policy

The authors declare that all data supporting the findings of this study are available within the article and its Supplementary Information files, or from the corresponding author upon reasonable request. The mass spectrometry proteomics data are available via ProteomeXchange with identifier PXD036285. Source data are provided with this paper.

Field-specific reporting

Please select the one below that is the best fit for your research. If you are not sure, read the appropriate sections before making your selection.

- [x] Life sciences
- [ ] Behavioural & social sciences
- [ ] Ecological, evolutionary & environmental sciences

For a reference copy of the document with all sections, see nature.com/documents/nr-reporting-summary-flat.pdf

Life sciences study design

All studies must disclose on these points even when the disclosure is negative.

Sample size	Sufficient sample sizes were chosen for each experiment to determine whether the outcome was statistically significant. The sample size in drug treatment or autophagy test followed some published papers and guidelines. At least two independent transgenic lines (from 10-15 positive transformation) were used for every phenotype study, and at least three repetitions were performed for every experiments with statical analysis.
Data exclusions	No data were excluded from this study.
Replication	We confirmed that all studies performed here is reproducible in all replications.
Randomization	All experimental samples were selected randomly.
Blinding	Blinding was not implemented in this study. No investigators in this study.

Reporting for specific materials, systems and methods

We require information from authors about some types of materials, experimental systems and methods used in many studies. Here, indicate whether each material, system or method listed is relevant to your study. If you are not sure if a list item applies to your research, read the appropriate section before selecting a response.

Materials & experimental systems

n/a	Involved in the study	
☐	☒	Antibodies
☒	☐	Eukaryotic cell lines
☒	☐	Palaeontology and archaeology
☐	☒	Animals and other organisms
☒	☐	Human research participants
☒	☐	Clinical data
☒	☐	Dual use research of concern

Methods

n/a	Involved in the study	
☒	☐	ChIP-seq
☒	☐	Flow cytometry
☒	☐	MRI-based neuroimaging

Antibodies

Antibodies used	Primary antibody: anti-TRB1 mouse, anti-VAP27 mouse, anti-RFP rat (Chromotek 5F8, Abcam ab62341), anti-GFP (Biorbyt orb323045);<br>Secondary antibody: FITC-conjugated mouse (Jackson ImmunoResearch 115-095-166), TRITC-conjugated rat (Jackson ImmunoResearch 112-025-167), HRP-conjugated mouse (Yeasen, 33201ES60)
Validation	All commercial antibodies were validated by the suppliers. VAP27 antibody was validated in previous publications, and TRB1 antibody is validated in this study.

Animals and other organisms

Policy information about studies involving animals; ARRIVE guidelines recommended for reporting animal research

Laboratory animals	Polyclonal antibodies were raised in 4-8 week old mice.
Wild animals	The study did not involve wild animals.
Field-collected samples	The study did not involve samples collected from the field.
Ethics oversight	Approval was granted by the project license holder and Animal Welfare and Ethical Review Board at Durham University.

Note that full information on the approval of the study protocol must also be provided in the manuscript.
