# Supplementary material for: TraB family proteins are components of ER-mitochondrial contact sites and regulate ER-mitochondrial interactions and mitophagy
Source: Nat Commun. 2022 Sep 26;13:5658. doi: 10.1038/s41467-022-33402-w (PMC9513094; doi:10.1038/s41467-022-33402-w)

Figure 1j

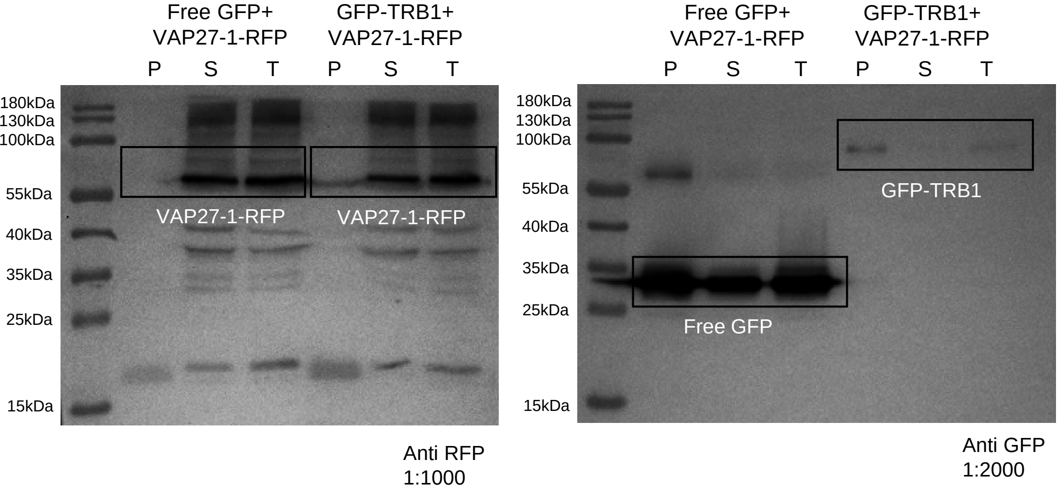

Figure 6o

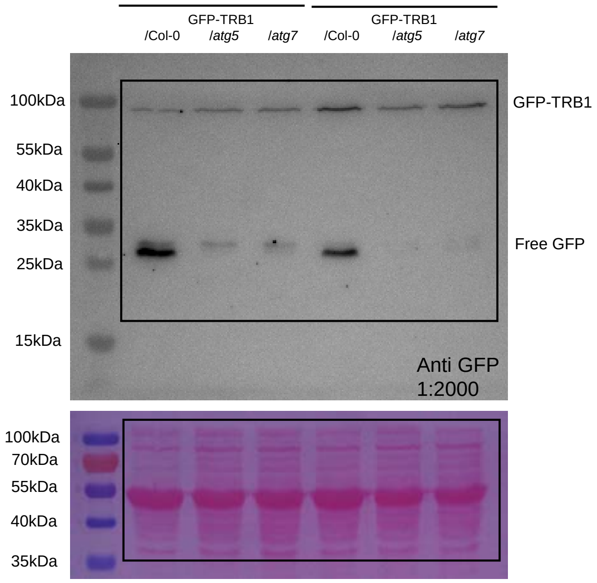

Figure 6q

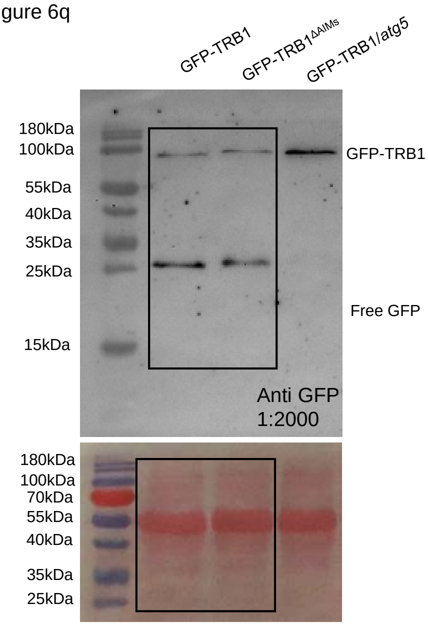

Figure 6o 2nd repeat

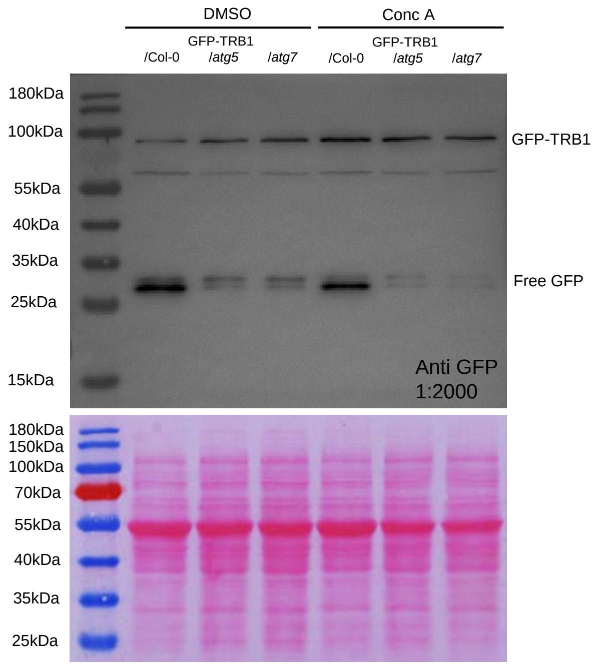

Figure 6q 2nd repeat

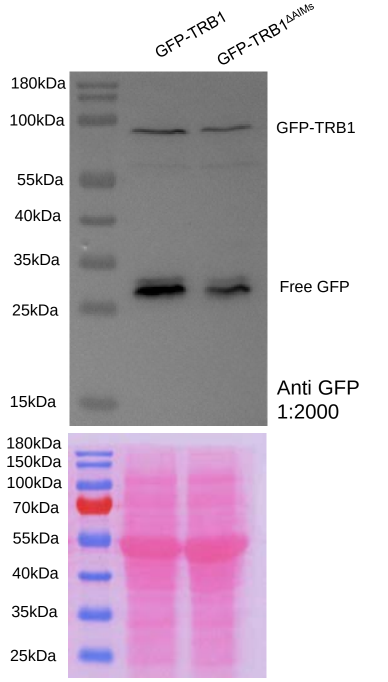

Supplementary Figures

Figure S2f

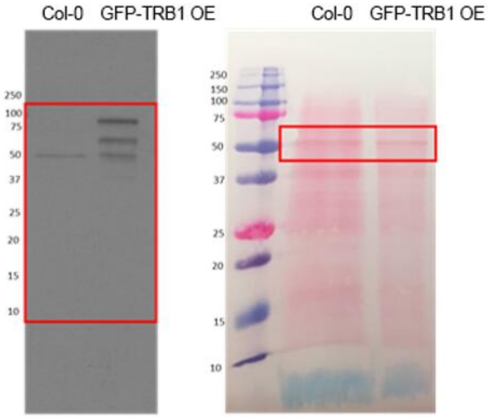

Figure S2g

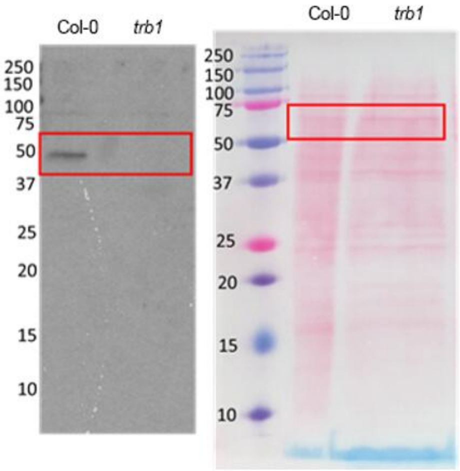

Figure S4b

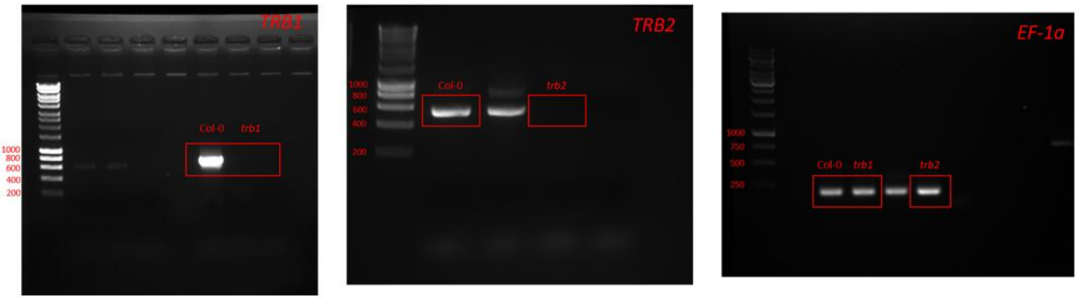

Supplement: Supplementary file 3 — Source Data [file 41467_2022_33402_MOESM3_ESM.zip › Li et al., Orignal Blot and Gel images.pdf]
